# Improved haplotype inference by exploiting long-range linking and allelic imbalance in RNA-seq datasets

**DOI:** 10.1038/s41467-020-18320-z

**Published:** 2020-09-16

**Authors:** Emily Berger, Deniz Yorukoglu, Lillian Zhang, Sarah K. Nyquist, Alex K. Shalek, Manolis Kellis, Ibrahim Numanagić, Bonnie Berger

**Affiliations:** 1 Computer Science & Artificial Intelligence Laboratory, Massachusetts Institute of Technology, Cambridge, MA 02139 USA; 2 Department of Mathematics, Massachusetts Institute of Technology, Cambridge, MA 02139 USA; 3 Department of Mathematics, UC Berkeley, Berkeley, CA 94720 USA; 4 Department of Computer Science, University of Victoria, Victoria, BC V8P 5C2 Canada

**Keywords:** Computational models, Data processing, Genome assembly algorithms, Sequence annotation, Software

## Abstract

Haplotype reconstruction of distant genetic variants remains an unsolved problem due to the short-read length of common sequencing data. Here, we introduce HapTree-X, a probabilistic framework that utilizes latent long-range information to reconstruct unspecified haplotypes in diploid and polyploid organisms. It introduces the observation that differential allele-specific expression can link genetic variants from the same physical chromosome, thus even enabling using reads that cover only individual variants. We demonstrate HapTree-X’s feasibility on in-house sequenced Genome in a Bottle RNA-seq and various whole exome, genome, and 10X Genomics datasets. HapTree-X produces more complete phases (up to 25%), even in clinically important genes, and phases more variants than other methods while maintaining similar or higher accuracy and being up to 10×  faster than other tools. The advantage of HapTree-X’s ability to use multiple lines of evidence, as well as to phase polyploid genomes in a single integrative framework, substantially grows as the amount of diverse data increases.

## Abstract (web summary)

Haplotype reconstruction of distant genetic variants is problematic in short-read sequencing. Here, the authors describe HapTree-X, a probabilistic framework that uses differential allele-specific expression to better reconstruct paternal haplotypes from diploid and polyploid genomes.

## Introduction

The two primary technologies for modern genetic association studies, genotyping arrays for common variants and next-generation sequencing for rare variants, are both limited to inferring only the genotype of an individual, but not in stitching these genetic differences into phased haplotypes^1^. This partial view can hide important interactions between nearby variants, and impede the search for understanding the molecular basis of human disease^2^. For instance, if an individual contains disease-risk variants in two different exons of the same gene, the genotype alone does not reveal whether both disease-associated mutations impact the same allele, thus leaving one functional copy, or whether they impact different alleles, leading to no functional copies of the gene. Such examples of compound heterozygosity have been associated with multiple diseases, including cerebral palsy, deafness, and haemochromatosis^3^. However, many additional examples likely remain undetectable given the lack of haplotype phasing information in the vast majority of disease association studies. The dearth of accurate haplotype phasing information can impact our ability to recognize optimal host-donor matches in organ transplantation, and also impede studies of human genetic variation, human population history reconstruction, ancestry determination for a given individual, and the study of genome evolution across individuals and across species^4^.

Methods for inferring phase information are traditionally based on pedigree information within large families^5,6^, but these apply mostly to traditional linkage studies and not to modern genome-wide association studies and rare variant sequencing studies, where relatedness is generally not known. More recently, large-scale population sequencing and genotyping studies such as HapMap^7^ and 1000 Genomes Project^2^ have provided experimentally phased or computationally phased reference genomes that can be used for phasing common variants^8–11^, but these maps are ineffective for de novo mutations or rare variants that are typically not well-represented, or accurately phased in these references.

More specialized computational methods for phasing operate on sequencing data alone and are able to phase rare and de novo mutations as they rely on sequencing reads that span two or more heterozygous SNPs^12–16^. However, many such methods are severely limited by the short sequence length for heterozygous SNP distances that exceed read fragment length. For some of these methods, speed and memory usage is also an issue^14,16^. Two exceptions—the recent proximity-ligation (Hi-C)^17^ and long-read sequencing (e.g., Pacific Biosciences or Oxford Nanopore) based methods^18,19^ that enable longer-range phasing—yet still require specialized technologies that are expensive and that suffer from high error rates. On the other hand, some high-throughput sequencing technologies—especially transcriptome sequencing via RNA-seq—are affordable, widely available, already established and standardized, and allow longer-range phasing within genes by leveraging the fact that the transcriptomic distance between SNPs may be less than the genomic distance.

**Fig. 1:**
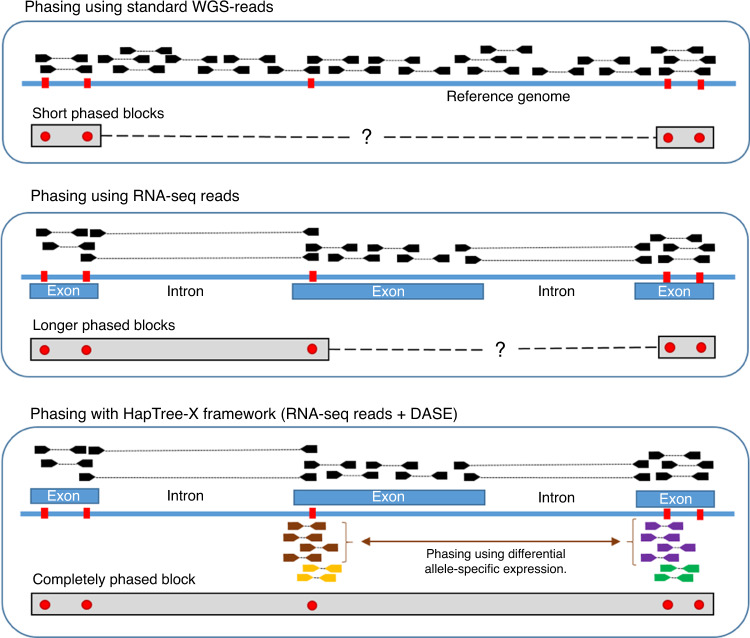
HapTree-X framework compared to read-based phasing. Traditional whole-genome sequencing (WGS) based phasing methods (top panel) depend on sequence contiguity and thus require a pair of SNPs (in red) to be connected through a common read that overlaps both in order to be phased. RNA-seq reads provide longer distance phasing capability due to long introns in the genome that are spliced-out in the sequenced transcript fragments (middle panel), yet SNPs that are far apart within the transcript due to long homozygous exonic regions are still difficult to phase using RNA-seq reads. Our HapTree-X framework (lower panel) overcomes this limitation by integrating RNA-seq reads and differential allele-specific expression (DASE) available from the RNA-seq data into a single probabilistic framework for haplotype phasing. For genes that display differential haplotypic expression (DHE), the majority of alleles can be phased together to obtain a single haplotype block for the entire gene. Depending on the DHE and depth-coverage, DASE-based phasing performs accurate haplotype reconstruction, without requiring paired-end or long reads, maintaining or improving on accuracy independent of gene/exon lengths as long as differential haplotypic expression is consistent across the loci being phased.

The splicing of RNA transcripts as they mature from pre-mRNAs to mRNAs provides an opportunity to mitigate the problem of short-read spans by bringing together exons across large genomic distances, thus enabling the recognition of heterozygous alleles that come from the same chromosomal copy^20,21^. However, these methods are still contiguity-based, relying on sequencing reads that span two or more heterozygous SNPs. Moreover, even the range of paired-end RNA-seq based phasing is limited by read fragment length in the presence of multiple or long intermediary exons that are devoid of heterozygous variants (Fig. 1). For instance, among the well-studied NA12878 transcripts that contain two or more heterozygous SNPs, one fifth contain a homozygous exonic region longer than 1000 bases between at least one pair of consecutive SNPs (Supplementary Note 1). Some attempts have been made to exploit underlying RNA-seq biases to improve the sequence-contiguity methods: examples include use of transcriptional bursting and technical dropout for haplotype phasing in single-cell RNA-seq datasets^17^; yet these signals are much less pronounced in classical RNA-seq data.

Here we introduce a conceptual advance that enables longer and more accurate haplotype phasing than existing sequence contiguity-based phasing methods for high-throughput RNA-seq datasets by tapping into the rich source of differential allele-specific expression (DASE) information within RNA-seq data. We follow the intuition that DASE in the transcriptome can be exploited to improve phasing because SNP alleles within maternal and paternal haplotypes of a gene are present in the read data at asymmetric frequencies due to the gene’s differential haplotypic expression (DHE). Phasing based upon differentially expressed allele frequencies additionally allows the use of reads covering only one heterozygous SNP, as opposed to existing methods which discard this information and rely solely on sequence contiguity (Fig. 1). Conceptually, given sufficient read coverage and DHE, all intra-genic SNPs of a gene with a single isoform can be phased using DASE, regardless of the transcriptomic distances between them. However, without knowing the underlying generative distributions of differential expression, we cannot extract linking information from this data source. We overcome this challenge by designing a Hidden Markov Model (HMM) to estimate the maximum likelihood underlying expression bias and prove that, with a few mild restrictions, the maximum likelihood estimate corresponds to concordant expression. We therefore present HapTree-X, an efficient and accurate phasing tool that performs single-individual haplotype reconstruction using RNA-seq data by exploiting DHE, in addition to spliced reads that overlap multiple variants. The core of the HapTree-X algorithm is the maximum likelihood framework that determines haplotype phasing by analyzing RNA-, DNA- or exome-seq and barcoded^22^ read data either independently or concurrently. HapTree-X enables long-range links to be used for phasing of much longer blocks. We demonstrate that our DASE-based portion leverages the large number of RNA-seq fragments that cover only one SNP—around an order of magnitude (more than 9×) more reads than other NGS-based methods can utilize, and increases the total phased block length up to 25% as compared to the other tools. We also show how DASE-based phasing of SNPs within genes with multiple isoforms can be theoretically achieved (Supplementary Note 3), with the restriction that the set of SNPs that can be phased is dependent on the composition and relative abundance of the multiple isoforms. HapTree-X generally decreases the switch error (SE) rate over the top-performing methods HapCUT^12^ and HapCUT2^15^—up to 15% in some cases—while at the same time phasing more SNPs and getting longer phased blocks; the commonly used SE rate is the percentage of positions where the two chromosomes of a phase must be switched in order to agree with the true phase when compared to a ground truth high confidence haplotype. On the other hand, methods with similar SE rates to HapTree-X^21^ phase orders of magnitude fewer SNPs and provide significantly shorter blocks than HapTree-X. We also show that HapTree-X provides more complete phases in many disease-related genes, and that it consistently phases longer clinically important genes better than other tools even on low-coverage datasets.

HapTree-X generalizes prior work, HapTree^14^—a maximum likelihood contig-based phaser that makes use of reads that span multiple SNPs—by non-trivially adapting the HapTree probabilistic model to now incorporate RNA-seq-specific priors that describe the correlation of allele-specific imbalance at SNP loci, allowing the construction of longer phased haplotype blocks. Furthermore, HapTree-X preserves the unique properties of HapTree, such as polyploid phasing, while adding capabilities such as incorporating long-range sequencing technologies^22^ and RNA-seq read data. HapTree-X also has greater scalability and is significantly faster than HapTree due to algorithmic and engineering improvements that reduce redundant computation as well as parallelization capabilities provided by the bioinformatics domain-specific language Seq^23^.

Not only does our general model readily integrate existing contiguity-based sequencing data that provides pairs of linked SNPs (e.g., Illumina whole-genome sequencing (WGS)^12^, exome sequencing, 10X long-range sequencing^22^, and RNA-seq without DASE^21^), it also is able to incorporate more complex diverse data as long as the user can give a reasonable prior about the underlying data; this usage case is demonstrated below where DASE-based phasing can phase reads covering only a single SNP.

## Results

### Datasets

We compared HapTree-X against state-of-the-art sequence-based computational phasing tools: HapCUT^12^, HapCUT2^15^, and phASER^21^. For benchmarking, we utilized the well-studied GM12878 sample, using cytosol, nucleus, and whole-cell RNA-seq data from the GM12878 lymphoblastoid cell-line from ENCODE CSHL Long RNA-seq track^24^, whole exome sequencing data from 1000 Genomes Project and a WGS sample from Illumina Platinum Genomes^25^; GENCODE release 19 was used as the reference gene annotation. We also included the K562 chronic myelogenous leukemia cell line RNA-seq data and validated it with the recently validated phasing ground truth dataset from ENCODE^26^. Lastly, we used five in-house sequenced Genome in a Bottle (GIAB)^27,28^ RNA-seq samples: NA12878, NA24143, NA24149, NA24385, and NA24631 and 10X Genomics’ publicly available GIAB samples and compared phased haplotype blocks to the gold-standard GIAB validation phases (Table 1). All RNA-seq samples were aligned with STAR aligner^29^ and genotyped by using GATK’s Best Practices workflow for RNA-seq data^30^. All phasers were run on a macOS desktop computer with 3.60 GHz Intel Core i9 CPU and 64 GB of RAM. For further details on the experimental setup, see Supplementary Note 2.

**Table 1:** Comparison of phasing quality for four different phasers: HapCUT, HapCUT2, phASER, and HapTree-X on 9 RNA-seq datasets with varying transcriptomic coverage and on four different RNA-seq datasets combined with the NA12878 exome dataset.

	HapCUT			HapCUT2			phASER^a^			HapTree-X		
GIAB (low coverage)												
NA12878	N/A			N/A			3,238	**0.95**	3387	**5871**	1.98	**6,927**
NA24143	N/A			N/A			2,399	**0.00**	3114	**5179**	**0.00**	**7,532**
NA24149	6,696	**0.97**	9,322	6,677	**0.97**	9,306	2,984	1.37	3125	**6710**	**0.97**	**9,414**
NA24385	7,079	1.75	8,100	7,055	1.86	7,971	3,896	**0.82**	3713	**7088**	1.64	**8,124**
NA24631	7,888	**0.00**	10,355	7,866	**0.00**	10,026	3,919	**0.00**	6303	**7892**	**0.00**	**10,414**
K562 leukemia cell line (medium coverage)												
K562	9993	0.96	4990	9972	0.82	3960	6770	**0.16**	2583	**10,270**	0.70	**6,819**
GM12878 (high coverage)												
Cytosol	28,706	2.61	18,724	28,699	2.62	18,441	14,451	**1.02**	11,846	**28,815**	2.59	**20,475**
Nucleus	31,420	2.23	21,249	31,418	2.23	21,208	17,377	**1.05**	13,137	**31,593**	2.19	**22,571**
Whole	30,520	1.91	18,960	30,520	1.89	18,960	15,420	**0.92**	10,932	**30,672**	1.94	**20,141**
NA12878 exome data (low coverage) with RNA-seq data												
GIAB	181,442	1.26	16,506	180,054	1.03	16,244	6188	1.55	5272	**181,467**	**1.01**	**16,787**
Cytosol	205,184	1.44	37,036	203,873	1.29	36,790	31,961	**1.20**	18,348	**205,483**	1.23	**41,643**
Nucleus	211,743	1.37	46,259	210,621	1.25	44,854	66,044	**0.93**	23,480	**212,214**	1.23	**50,763**
Whole	209,252	1.31	37,773	208,060	1.15	37,375	54,475	**0.95**	17,039	**209,694**	1.15	**42,624**

Cells contain the number of SNPs phased, switch error (SE) rate, and total length of phased blocks (span) in kilobases by a phaser for a dataset. Bold values represent the best overall results for a metric in the dataset. Overall, HapTree-X consistently phases more SNPs with comparable or lower switch error rates and longer phased blocks.

*N/A* a tool was not able to successfully complete the phasing.

^a^phASER, as a rule, uses more stringent filtering and thus achieves lower switch rate while phasing order of magnitude less SNPs than the other tools; however, HapTree-X’s SE rates are comparable if we restrict it to the same phasing blocks.

### RNA-seq results

The results in Table 1 show that HapTree-X generally decreases the SE rate over HapCUT and HapCUT2—up to 15% in some cases—while at the same time phasing more SNPs and getting up to 25% longer phased blocks, as in the K562 leukemia cells. While phASER has overall lower SE rate, this is due to its phasing an order of magnitude less SNPs because of stringent block filtering as compared to the other tools. However, when restricted to phASER’s blocks, the difference in SE either disappears or becomes negligible: in the worst case, HapTree-X introduces no more than 15 SEs over the 7000 validated phased SNPs (causing the effective error rate to be less than 0.2%).

### Other technologies

HapTree-X is also able to use RNA-seq data to improve phasing of classical DNA sequencing. Table 1 shows that HapTree-X can increase the span of phasing blocks up to 12% in joint exome and RNA-seq data while maintaining lower SE over HapCUT and HapCUT2 (the aforementioned observation about phASER results still applies). HapTree-X also phases and links up to 500 SNPs ignored by other phasers. On WGS datasets, HapTree-X outperformed HapCUT2 both in terms of SE rate and runtime; we also observed a total phased block length increase of 30% in the joint WGS and RNA-seq experiment (Table 2).

**Table 2:** Comparison of HapCUT2 and HapTree-X (single-threaded mode) on WGS and 10X Genomics datasets.

	HapCUT2		HapTree-X	
NA12878 whole-genome sequencing (WGS)	1:38:38	(16.21)	**0:38:04**	(**16.12**)
NA12878 WGS with nucleus RNA	1:48:01	(16.41)	**0:40:17**	(**15.20**)
10X Genomics NA12878	22:07:05	(1.11)	**1:54:05**	(**1.09**)
10X Genomics NA24385	22:13:43	(4.83)	**1:53:16**	(**4.81**)

Cells contain runtime and switch error rate (in parenthesis). Bold values represent the best overall results for a metric in the dataset. HapTree-X is from 3 to 10× faster than HapCUT2 while providing better or comparable switch rates. Time units are in h:mm:ss.

We compared HapTree-X to HapCUT2 (the only state-of-the-art phaser that can phase 10X data) not only on a whole-genome Platinum NA12878 dataset, but also on two high coverage 10× datasets that were aligned by the EMA aligner^31^ (Table 2). HapTree-X was able to phase much faster than HapCUT2 (with up to 10×  speed-up) while maintaining overall better switch rate.

Finally, we note that the polyploid capabilities of HapTree-X are identical to those of HapTree (except that the new pipeline is computationally more efficient). For these reasons, we refer readers to the original HapTree results for polyploid phasing^14^.

### Performance and usability

In addition to accurate results, we demonstrate significant speed improvements over other phasing methods tested (Table 3)—HapTree-X is often twice as fast as HapCUT2, and in the case of 10× data, HapTree-X is more than 10× faster. HapTree-X is also the only phaser that can use more than one thread to perform phasing: while even in single-threaded mode HapTree-X is the fastest phaser, by using four threads HapTree-X runs even faster, allowing the user to complete joint exome and RNA-seq analysis in 5 min or less, and to complete joint WGS and RNA-seq analysis (≈180 GB of data) in less than 25 min. Note that the runtime of HapTree-X is negligible as compared to the best practices genotyping pipeline (which takes at least a day to complete on a cluster).

**Table 3:** Comparison of runtime between different phasing tools (in format (h):mm:ss) on a few representative samples (all other samples display the similar ratios between runtimes).

	HapCUT	HapCUT2	phASER	HapTree-X	HapTree-X (4 threads)
GIAB (NA24149)	3:03	1:30	1:08	**0:54**	0:27
GM12878 (Nucleus)	21:10	12:59	16:55	**8:59**	3:15
Exome (Whole)	31:21	17:13	35:03	**12:22**	5:10
Exome (Cytosol)	25:46	12:57	24:23	**8:59**	3:36
WGS (Nucleus)	N/A	1:48:01	N/A	**40:17**	23:16
10X (NA12878)	N/A	22:07:05	N/A	**1:54:05**	57:37

Bold values represent the fastest runtime in single-threaded mode on a dataset. HapTree-X is clearly the fastest phaser, being up to 10× faster than the fastest competitor. N/A indicates that the tool was not evaluated on that sample.

HapTree-X can be added downstream to any pre-existing RNA-seq processing pipeline to output phased haplotype blocks. HapTree-X takes as input RNA-seq read alignment files (SAM/BAM format), a standard VCF file containing the individual’s genotype, and a gene annotation that specifies the boundaries of genes and their exons. Finally, we note that HapTree-X can easily incorporate different technologies during the phasing.

### Effect of DASE

Incorporating DASE into phasing enables HapTree-X to increase the number of phased SNPs and the length of phased blocks within genes in RNA-seq data. While DASE had modest impact on low-coverage GIAB samples, increasing the total phase length by only 1%, increased coverage on GM12878 samples caused DASE to increase the total phase length to 5% over other tools and HapTree-X with DASE turned off. The DASE effect is much stronger in joint exome and RNA-seq analysis: we observed up to a 12% increase in total phase length. We noticed that DASE performs the best on the K562 leukemia cell line, where the total phase length went up by 25%. We provide a theoretical explanation of this effect by showing that accuracy increases exponentially with FPKM depth-coverage—fragments per kilobase of transcript per million mapped reads (Supplementary Note 1, and Supplementary Figs. 1 and 2). As the cost of RNA-seq data decreases, datasets with increasing coverage will become more accessible, substantially expanding the impact of HapTree-X. Finally, we note that DASE itself is responsible for inclusion of many SNPs that are otherwise excluded by HapTree and other tools—in the case of combined RNA-exome datasets, DASE is able to use and link up to 1000 previously unphased SNPs as compared to HapTree-X without DASE. In a few cases, more SNPs result in slightly increased switch error (SE) rate as compared to HapTree-X without DASE and other tools. We examined those errors, and found that the number of SNPs that one needs to remove to achieve better SE rates is an order of magnitude less than the number of SNPs that are additionally phased.

### HapTree-X improves phasing in clinically significant genes

**Fig. 2:**
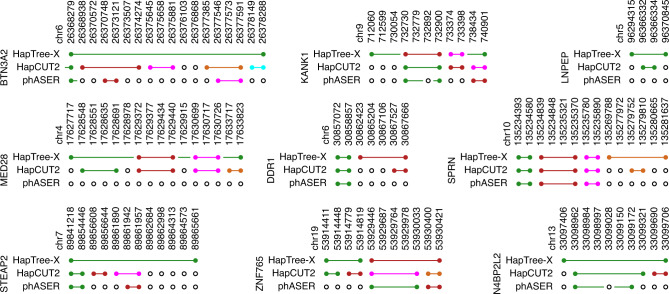
Phasing of nine disease-associated genes by HapTree-X, HapCUT2, and phASER using whole-cell RNA-seq data from GM12878. Unphased SNPs are represented by an empty circle, and each phased block is given a unique color. Note that some blocks might overlap because not all SNPs from a gene exhibit DASE. Reported SNP loci are relative to the human genome hg19 (GRCh37).

HapTree-X links SNP pairs in the GM12878 dataset that could not be phased by sequence contiguity-based methods. Such phased SNPs enable us to better phase genes that have clinical associations with various diseases; a few significant examples that show *BTN3A2* (associated with epithelial ovarian cancer^32^), *KANK1* (cerebral palsy^33^), *LNPEP* (autism spectrum disorders^34^), *MED28* (breast cancer^35^), *DDR1* (schizophrenia^36^), *SPRN* (Creutzfeldt-Jakob disease^37^), *STEAP2* (prostate cancer^38^), *ZNF765* (renal cell carcinoma^39^), and *N4BP2L2* (arsenic poisoning^40^) genes are shown in Fig. 2 (note that this list is not exhaustive: we just selected a few genes to illustrate the improvements by HapTree-X). HapTree-X not only phases previously unphased SNPs, but can also link separate blocks found by other methods and thus give more complete phasing results that link all the heterozygous SNPs in these genes (Fig. 2).

**Fig. 3:**
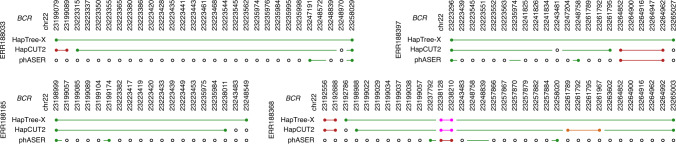
Phasing of the *BCR* gene by HapTree-X, HapCUT2, and phASER on a selection of four GEUVADIS RNA-seq samples. Unphased SNPs are represented by an empty circle, and each phased block is given a unique color. Reported SNP loci are relative to the human genome hg19 (GRCh37).

To demonstrate that these improvements are not individual-specific, we ran HapTree-X and other tools on thirty 1000 Genomes GEUVADIS RNA-seq samples^41^. All of these samples were low-coverage RNA-seq samples, and thus could not benefit from DASE as much as GM12878 samples. Nevertheless, HapTree-X phased more SNPs in all cases than the other methods, and DASE consistently (17 out of 30 samples) improved phasing of the long *BCR* gene, which has a causal relationship to chronic myeloid leukemia^42,43^ (see Fig. 3 for the illustration of these improvements).

## Discussion

With improvements in sequencing technologies, the ability to capitalize on diverse and available sequencing data will become critical to fully realizing the potential of large-scale genomics. The HapTree-X software provides joint DNA and RNA phasing capability that achieves better phasing performance than either data source used alone. It leverages the long-range phasing capabilities of RNA-seq and DASE to increase the span and completeness of regions phased with read overlap information. This enhances the phasing of even noncoding and non-expressed regions of the genome when used in combination with genome or exome sequencing datasets. As such, it can be incorporated as a pre- or post-processing step in conjunction with existing population-based phasing pipelines to provide more complete phases.

While linked read-based phasing technologies show great promise for long-range phasing applications (and HapTree-X can use this data as shown with 10×), RNA-seq datasets are currently cheaper, more prevalent and contain abundant long-range phasing information via splicing and DASE that is currently underutilized. Notwithstanding the inherent limitations of RNA-seq data that reduce the scope of DASE-enabled optimizations, such as the small transcriptome size and gene-restricted phases, we show that such data still harbors enough valuable information to significantly improve phasing quality of both RNA-only and joint DNA-RNA analyses, with no impact on the computational resources.

In the near future, we plan to extend HapTree-X to single-cell RNA-seq datasets that are rapidly becoming more affordable and common^44,45^. We also expect to see further validation of HapTree-X’s theoretical framework as the coverage of RNA-seq and the size of the ground truth datasets expand: DASE phases better if the coverage is higher, and the large portion of SNPs phased by any of the evaluated tools are not currently validated by the GIAB project (as HapTree-X phases the largest number of SNPs, we expect it to benefit the most from the more complete validation sets). Finally, we are looking to expand our DASE theoretical framework to other problems, as other kinds of data—such as barcoded reads—exhibit similar biases that can be in principle modeled by the same theoretical framework.

The fast access to more-comprehensively phased gene regions opens the door for further understanding of the relationship between genotype and phenotype in biomedical disease research. Our conceptual advance, as well as our implementation, will greatly benefit researchers who analyze large amounts of DNA and RNA sequencing data, regardless of the technology.

## Methods

### Overview of HapTree-X

HapTree-X is a Bayesian haplotype reconstruction framework which simultaneously employs read overlap information (through read contiguity or read barcodes) and optional DASE for haplotype phasing. HapTree-X outputs phased haplotype blocks, given an input of read alignment files (BAM/SAM), a VCF file containing the individual’s genotype, and an optional gene model which specifies the genes (and their exons) within the genome. It is able to take multiple lines of evidence (e.g., both RNA and DNA-seq aligned files) at the same time for improved phasing.

The HapTree-X pipeline is initiated by determining which genes are expressed using the gene model and RNA-seq data. For each of these genes, a maximum likelihood expression bias (DHE) is computed. Furthermore, we determine which SNPs within those genes have high likelihood of concordant expression; we phase only those SNPs. For reads containing only such SNPs, we assign to them the computed expression bias of the gene they cover; for all other reads, we assign a non-biased expression. Finally, applying a generalized version of HapTree^14^, we determine a haplotype of maximal likelihood which depends on the DASE present in the RNA-seq data, as well as the sequence-contiguity information within the reads.

### A high-level overview of the DASE-based phasing

Using DASE for phasing presents major challenges. Consider a simple example, presented in Table 4, where we have a single gene, no splicing, and each read covers one SNP. We can attempt to phase the gene using DASE. If we already knew that the DHE was *β* = 0.9, then it would be straightforward to guess the haplotypes as in Table 4.

**Table 4:** A toy phasing example on five SNPs: the counts of mutant/reference allele observations for each SNP (left) and the inferred haplotypes (right), assuming that the differential haplotype expression was *β* = 0.9.

Allele/SNP	1	2	3	4	5		Allele/SNP	1	2	3	4	5
Reference	12	15	79	97	11	⟶	Reference	0	0	1	1	0
Mutant	92	85	7	4	84		Mutant	1	1	0	0	1

However, we overcome the difficulty that the underlying DHE is unknown, often not as drastically high as *β* = 0.9, and must instead be inferred from the same expression data. Furthermore, the integration of these data with reads covering multiple SNPs, as well as the complications arising from multiple genes and splicing makes this inference highly nontrivial.

We present a Bayesian mathematical framework for estimating ${\mathcal{B}}$, which allows inference of long-range haplotype links, using a combination of HMMs and maximum likelihood analysis (Online Methods). Our framework seeks to determine the haplotype of maximal probability given the observed read data (*R*), DHE (${\mathcal{B}}$), and error rates (*ε*). Applying Bayes’ rule, we can reduce this problem to determining the haplotype *H* which maximizes the product over all reads *R* of the probability of observing each read *r*, given *H* is the true haplotype:

1 $${\rm{P}}[H| R,{\mathcal{B}},\varepsilon ]=\frac{{\rm{P}}[R| H,{\mathcal{B}},\varepsilon ]{\rm{P}}[H| {\mathcal{B}},\varepsilon ]}{{\rm{P}}[R| {\mathcal{B}},\varepsilon ]}\,{\rm{where}}\,{\rm{P}}[R| H,{\mathcal{B}},\varepsilon ]=\prod_{r\in R}{\rm{P}}[r| H,{\mathcal{B}},\varepsilon ].$$

To compute this probability, for each read *r*, we partition the SNPs covered by *r* into *A*(*r*, *H*_*i*_) and *D*(*r*, *H*_*i*_) (those SNPs where the read *r* and haplotype *H* agree and disagree, respectively) and take the product of the probabilities of agreement and disagreement, along with the assumed rate of expression (see below for further context and details of notation):

2 $${\rm{P}}[r| H,{\mathcal{B}},\varepsilon ]={\sum }_{i\in [0,1]}\left({\beta }_{i}^{r} \prod_{s\in A(r,{H}_{i})}(1-{\varepsilon }_{r,s})\prod_{s\in D(r,{H}_{i})}{\varepsilon }_{r,s}\right).$$

### Notation

The goal of phasing is to recover the unknown haplotypes (haploid genotypes), *H* = (*H*_0_, *H*_1_), which contain the sequence of variant alleles inherited from each parent of the individual. As homozygous SNPs are irrelevant for phasing, we restrict ourselves to heterozygous SNPs (from now on referred to simply as an SNP) and we denote the set of these SNPs as *S*. We assume these SNPs to be biallelic, and because of these restrictions, *H*_0_ and *H*_1_ may be expressed as binary sequences, where a 0 denotes the reference allele and a 1 the alternative allele; *H*_0_ and *H*_1_ are complement sequences. Let *H*[*s*] = (*H*_0_[*s*], *H*_1_[*s*]) denote the alleles present at *s*, for *s* ∈ *S*.

We denote the sequence of observed nucleotides of a fragment simply as a read (independent from single/paired-end reads). We assume each read is mapped accurately and uniquely to the reference genome, and moreover that each read is sampled independently (note that the problem of multi-mappings in RNA-seq data should be resolved upstream of the HapTree-X pipeline with the tools such as ORMAN^46^). The set of all reads is denoted as *R*. Given a set of SNP loci *S*, we define a read *r* ∈ *R* as a vector with entries *r*[*s*] ∈ {0, 1, −}, for *s* ∈ *S*, where a 0 denotes the reference allele, a 1 the alternative allele, and  −  that the read does not overlap *s* or that it contains an allele that is not observed in the genotype of locus *s* (likely due to a sequencing error). We say a read *r* ∈ *R* contains an SNP *s* if *r*[*s*] ≠ −  and we let size of a read *r*, ∣*r*∣, refer to the number of SNPs it contains. For each read *r* and for each SNP locus *s*, we assume a probability of opposite allele information *r*[*s*] equal to *ε*_*r*,*s*_ and represent these error probabilities as a matrix *ε*. We assume these errors to be independent from one another. (Note that we model opposite allele errors here, and not SEs: SE is merely a commonly used accuracy measure for the quality of properly estimating opposite allele errors.)

In genomic read data, all *r* ∈ *R* are equally likely to be sampled from the maternal or paternal chromosomes. In RNA-seq data however, this may not always be the case. In this paper, we define the DHE to represent the underlying expression bias between the maternal and paternal chromosomes of a particular gene. Throughout, we will refer to the probability of sampling from the higher frequency haplotype of a gene as *β*. We assume two genes $g,g^{\prime}$ have independent expression biases $\beta, \beta ^{\prime}$. DASE we define as the observed bias in the alleles at a particular SNP locus present in *R*. We define the event of concordant expression to be when the DASE of an SNP agrees with the DHE of the gene to which the SNP belongs. To perform phasing using the sequence contiguity within reads (contig-based phasing), upon the set of SNP loci *S* and read set *R*, we define a read graph such that there is a vertex for each SNP locus *s* ∈ *S* and an edge between any two vertices $s,s^{\prime}$ if there exists some read *r* containing both *s* and $s^{\prime}$. These connected components correspond to the haplotype blocks to be phased.

To phase using differential expression (DASE-based phasing), we assume the existence of some gene annotation *G* that specifies the genes (and their exons) within the genome. We used GENCODE v19 annotation for our experiments on NA12878. For each *g* ∈ *G*, we assume that the haplotypes (*H*_0_, *H*_1_) restricted to *g* are expressed at rates *β*_0_, *β*_1_ respectively due to DHE. The phasing blocks correspond to the SNPs in genes *g* ∈ *G*, though we will see that some SNPs are not phased due to insufficient probability of concordant expression. Two distinct genes $g,g^{\prime}$ may not be DASE-phased due to lack of correlation between their expression biases $\beta ,\beta ^{\prime}$. In the remainder of this paper, when DASE-phasing a particular gene, by *H* we mean the gene haplotype, that is *H* restricted to the SNPs within *g*.

The final blocks to be phased by HapTree-X integrating both contig and DASE-based phasing are defined as the connected components of a joint read graph. The vertices are the SNPs phased by either method, and there is an edge between any two $s,s^{\prime}$ if there exists some block (from either method) containing both $s,s^{\prime}$.

### Likelihood of a phase

We formulate the haplotype reconstruction problem as identifying the most likely phase(s) of set of SNPs *S*, given the read data *R*, and sequencing error rates *ε*. Furthermore, suppose we knew for each read *r*, the likelihood that *r* was sampled from *H*_*i*_ (denote this as ${\beta }_{i}^{r}$); we represent these probabilities as a matrix ${\mathcal{B}}$. While ${\mathcal{B}}$ is not given to us, we may estimate ${\mathcal{B}}$ from *R*. We derive a likelihood equation for *H*, conditional on $R,{\mathcal{B}}$ and *ε*.

Given a haplotype *H*, reads *R*, error rates *ε*, and expression rates ${\mathcal{B}}$, the likelihood of *H* being the true phase is given by

3 $${\rm{P}}[H| R,{\mathcal{B}},\varepsilon ]=\frac{{\rm{P}}[R| H,{\mathcal{B}},\varepsilon ]{\rm{P}}[H| {\mathcal{B}},\varepsilon ]}{{\rm{P}}[R| {\mathcal{B}},\varepsilon]}.$$

Since ${\rm{P}}[R| {\mathcal{B}},\varepsilon ]$ does not depend on *H*, we may define a relative likelihood measure, RL. Note that ${\rm{P}}[H| {\mathcal{B}},\varepsilon ]={\rm{P}}[H]$ as the priors on the haplotypes are independent of the errors in *R*, and of ${\mathcal{B}}$.

4 $${\rm{RL}}[H| R,{\mathcal{B}},\varepsilon ]={\rm{P}}[R| H,{\mathcal{B}},\varepsilon ]{\rm{P}}[H].$$

For the prior P[*H*], we assume a potential parallel bias, *ρ* ≥ 0.5, which results in a distribution on *H* such that adjacent SNPs are independently believed to be phased in parallel (00) or (11) with probability *ρ* and switched (01) or (10) with probability 1 − *ρ*. When *ρ* = 0.5 we have the uniform distribution on *H*. The general prior distribution on *H* in terms of *ρ* is

5 $${\rm{P}}[H]={\rho }^{P(H)}{(1-\rho )}^{S(H)},$$

where *P*(*H*) and *S*(*H*) denote the number of adjacent SNPs that are parallel and switched in *H*, respectively. Given the above model, as each *r* ∈ *R* independent, we may expand ${\rm{P}}[R| H,{\mathcal{B}},\varepsilon ]$ as a product:

6 $${\rm{P}}[R| H,{\mathcal{B}},\varepsilon ]=\prod_{r\in R}{\rm{P}}[r| H,{\mathcal{B}},\varepsilon ]$$

In the setting of RNA-seq, reads are not sampled uniformly across homologous chromosomes, but rather according to the DHE (expression bias) of the gene from which they are transcribed. We see in Eq. (7) how this asymmetry allows us to incorporate reads which contain only one SNP. Let *A*(*r*, *H*_*i*_), *D*(*r*, *H*_*i*_) denote the SNP loci where *r* and *H*_*i*_ agree and disagree respectively; then it follows that

7 $${\rm{P}}[r| H,{\mathcal{B}},\varepsilon ]=\sum_{i\in [0,1]}{\beta }_{i}^{r}\prod _{s\in A(r,{H}_{i})}(1-{\varepsilon }_{r,s})\prod_{s\in D(r,{H}_{i})}{\varepsilon }_{r,s}.$$

When there is uniform expression ${\beta }_{0}^{r}={\beta }_{1}^{r}$ (no bias) and if ∣*r*∣ = 1, then ${\rm{P}}[r| H,{\mathcal{B}},e]$ is constant across all *H*. This is not the case when the expression bias is present however, and therefore reads covering only one SNP affect the likelihood of *H*.

If we knew the matrix ${\mathcal{B}}$, we could apply HapTree to search for *H* of maximal likelihood; the matrix ${\mathcal{B}}$, however, is unknown. Suppose instead we are given some probability distribution for the entries of ${\mathcal{B}}$, to compute ${\rm{P}}[r| H,{\mathcal{B}},\varepsilon ]$, it is enough to know the expected value of each entry because of the linearity (over *i*) of ${\rm{P}}[r| H,{\mathcal{B}},\varepsilon ]$. To this aim, we provide methods for determining a maximum likelihood ${\mathcal{B}}$. To approximate distributions for the entries of ${\mathcal{B}}$, we assume for each gene there is uniform expression with some probability *p*, and differential expression with probability 1 − *p*; in the latter case, the differential expression is assumed to be that of maximal likelihood. By varying *p*, we can vary the relative weights associated to DASE-based phasing and contig-based phasing. Furthermore, we develop methods for determining for which reads *r* we are sufficiently confident there this is in fact non-uniform expression, that is ${\beta }_{0}^{r} \, \ne \, {\beta }_{1}^{r}$. Moreover, we determine for which SNPs *s* ∈ *S* (contained only by reads of size one), we have sufficient coverage and expression bias to determine (with high accuracy) the phase *H*[*s*].

### Maximum likelihood estimate of DHE

For a fixed gene *g*, containing SNPs *S*_*g*_, the corresponding reads *R*_*g*_ have expression biases ${\beta }_{0}^{r},{\beta }_{1}^{r}$ which are constant across *r* ∈ *R*_*g*_. Let $\beta ={\beta }_{0}^{r}$ refer to this common expression; we wish to determine the maximum likelihood underlying expression bias *β* of *g* responsible for producing *R*_*g*_. To do so, we formulate an HMM and use the forward algorithm to compute relative likelihoods of *R* given *β*, *ε*.

To achieve the conditional independence required in an HMM, we define $${R}_{g}^{\prime}$$, a modification of *R*_*g*_, containing only reads of size one, so that $${R}_{g,s}^{\prime}$$ (the reads $$r\in {R}_{g}^{\prime}$$ which cover *s*) are independent from $${R}_{g,s^{\prime} }^{\prime}$$$$(\forall s \, \ne \, s^{\prime} \in {S}_{g})$$. We restrict each *r* ∈ *R*_*g*_ to a uniformly random SNP *s*, and include this restricted read of size one (*r*∣_*s*_) in $${R}_{g}^{\prime}$$ (we note that if ∣*r*∣ = 1, then *r* = *r*∣_*s*_, by definition.) Therefore, $${R}_{g,s}^{\prime}$$ and $${R}_{g,s^{\prime} }^{\prime}$$ are independent as all $$r\in {R}_{g}^{\prime}$$ are of size one.

Our goal is to determine the maximum likelihood *β*, given ${R}_{g}^{\prime}$. We assume a uniform prior on *β*, and therefore ${\rm{P}}[\beta | {R}_{g}^{\prime},\varepsilon ]$ is proportional to ${\rm{P}}[{R}_{g}^{\prime}| \beta ,\varepsilon ]$ (immediate from Bayes theorem). We may theoretically compute ${\rm{P}}[{R}_{g}^{\prime}| \beta ,\varepsilon ]$ by conditioning *H* (which is independent from *β*, *ε*)

8 $${\rm{P}}[{R}_{g}^{\prime}| \beta ,\varepsilon ]=\sum_{H}{\rm{P}}[{R}_{g}^{\prime}| H,\beta ,\varepsilon ]{\rm{P}}[H],$$

and expand ${\rm{P}}[{R}_{g}^{\prime}| H,\beta ,\varepsilon ]$ as a product over $r\in {R}_{g}^{\prime}$ as in Eqs. (6) and (7). This method, however, requires enumerating all *H*; since $| H| ={2}^{| {S}_{g}| }$ we seek different approach. Indeed, we translate this process into the framework of an HMM, apply the forward algorithm to compute $f(\beta ):= {\rm{P}}[{R}_{g}^{\prime}| \beta ,\varepsilon ]$ exactly for any *β*, and since *f* has a unique local maxima for *β* ∈ [0.5, 1], we can apply Newton-Rhapson method to determine *β* of maximum likelihood.

To set this problem in the framework of an HMM, we let the haplotypes *H* correspond to the hidden states, ${R}_{g}^{\prime}$ to the observations, and let the time evolution be the ordering of the SNPs *S*_*g*_. The observation at time *s* in this context is ${R}_{g,s}^{\prime}$, the reads covering SNP *s*. The emission distributions are as follows:

9 $${\rm{P}}[{R}_{g,s}^{\prime}| H[s],\beta ,\varepsilon ]=\prod_{r\in {R}_{g,s}^{\prime}}{\rm{P}}[r| H[s],\beta ,\varepsilon ],$$

10 $${\rm{P}}[r| H[s],\beta ,\varepsilon ]=\left\{\begin{array}{ll}{\beta }_{0}(1-{\varepsilon }_{r,s})+(1-{\beta }_{0}){\varepsilon }_{r,s};&r[s]={H}_{0}[s]\\ {\beta }_{1}(1-{\varepsilon }_{r,s})+(1-{\beta }_{1}){\varepsilon }_{r,s};&r[s]={H}_{1}[s]\end{array}\right.,$$

where *H*[*s*] is *H* restricted to *s*.

To determine the hidden state transition probabilities, recall our prior on *H* in Eq. (5). We may equivalently model this distribution *H* as a Markov chain, with transition probabilities:

11 $${\rm{P}}[H[{s}_{i+1}]| H[{s}_{i}]]=\left\{\begin{array}{ll}{\hskip -16pt}\rho &\,\,\,\,{\text{if}}\,{H}_{0}[{s}_{i}]={H}_{0}[{s}_{i+1}]\\ 1-\rho &\,\,{\text{if}}\,{H}_{0}[{s}_{i}] \,\, \ne \,\, {H}_{0}[{s}_{i+1}]\end{array}\right.$$

These emission probabilities and hidden state transition probabilities are all that are needed to apply the forward algorithm and determine the *β* of maximum likelihood.

### Likelihood of concordant expression

Here we prove that the intuitively correct solution (under mild conditions) is that of maximal likelihood. In doing so, we see the role played by concordant expression, and motivate its use as a probabilistic measure for determining which SNPs we believe we may phase with high accuracy.

Under a certain set of conditions, we derive *H*^+^, a haplotype solution of a gene *g*, of maximum likelihood given ${R}_{g}^{\prime}$, *β* and *ε*. Let ${C}_{s}^{v}$ denote the number of reads $r\in {R}_{g,s}^{\prime}$ such that *r*[*s*] = *v* where *v* ∈ {0, 1}. Provided error rates are constant (say *ϵ*) and *ϵ* < 0.5, and assuming a uniform prior distribution (*ρ* = 0.5), we can show a solution of maximum likelihood is ${H}^{+}=({H}_{0}^{+},{H}_{1}^{+})$, where ${H}_{0}^{+}[s]=v$ such that ${C}_{s}^{v}\ge {C}_{s}^{1-v}$. In words, ${H}_{0}^{+}$ and ${H}_{1}^{+}$ contain the alleles that are expressed the majority and minority of the time (respectively) at each SNP locus; given sufficient expression bias and coverage, intuitively, *H*^+^ ought to correctly recover the true haplotypes.

To prove *H*^+^ is of maximal likelihood, we introduce the terms concordant expression and discordant expression. We say *R* and *H* have concordant expression at *s* if ${C}_{s}^{{H}_{0}[s]} \, > \, {C}_{s}^{{H}_{1}[s]}$, discordant expression if ${C}_{s}^{{H}_{0}[s]} \, < \, {C}_{s}^{{H}_{1}[s]}$, and equal expression otherwise. In words, since we assume *β*_0_ > *β*_1_, we expect to see the allele *H*_0_[*s*] expressed more than the allele *H*_1_[*s*] in *R*_*g*,*s*_ (concordant expression).

We may now equivalently define *H*^+^ as a solution which assumes concordant or equal expression at every SNP *s*. Because we assume uniform priors, ${\rm{P}}[H| {R}_{g}^{\prime},\beta ,\epsilon ]$ is proportional ${\rm{P}}[{R}_{g}^{\prime}| H,\beta ,\epsilon ]$ (see Eq. (3)), and since each read is of size one, we can factor across *S*_*g*_ in the following way:

12 $$p[R| H,\beta ,\epsilon ]=\prod _{s\in {S}_{g}}{\rm{P}}[{R}_{g,s}| H[s],\beta ,\epsilon ].$$

Therefore, to show *H*^+^ is of maximal likelihood, it only remains to show that concordant expression is at least as likely as discordant expression, as intuition suggests. Let *γ*_*i*_ = *β*_*i*_(1 − *ϵ*) + (1 − *β*_*i*_)*ϵ*, then as in Eq. (7) we may deduce

13 $${\rm{P}}[{R}_{g,s}| H[s],\beta ,\epsilon ]=\prod _{i\in \{0,1\}}{\gamma }_{i}^{{C}_{s}^{{H}_{i}[s]}}.$$

Let ${H}^{-}=({H}_{1}^{+},{H}_{0}^{+})$, the opposite of *H*^+^. We can now compare the likelihood of concordant (or equal) expression at *s*(*H*^+^[*s*]) with that of discordant (or equal) expression at *s* (*H*^−^[*s*].) For ease of notation, let ${v}_{i}={H}_{i}^{+}[s]$ and ${w}_{i}={H}_{i}^{-}[s]$. Then:

14 $$\frac{{\rm{P}}[{R}_{g,s}| {H}^{+}[s],\beta ,\epsilon ]}{{\rm{P}}[{R}_{g,s}| {H}^{-}[s],\beta ,\epsilon ]}=\frac{{\prod }_{i\in \{0,1\}}{\gamma }_{i}^{{C}_{s}^{{v}_{i}}}}{{\prod }_{i\in \{0,1\}}{\gamma }_{i}^{{C}_{s}^{{w}_{i}}}}=\frac{{\gamma }_{0}^{{C}_{s}^{{v}_{0}}-{C}_{s}^{{w}_{0}}}}{{\gamma }_{1}^{{C}_{s}^{{w}_{1}}-{C}_{s}^{{v}_{1}}}}={\left(\frac{{\gamma }_{0}}{{\gamma }_{1}}\right)}^{{C}_{s}^{{v}_{0}}-{C}_{s}^{{v}_{1}}}\ge 1$$

The rightmost equality results from the fact that ${H}_{i}^{+}={H}_{1-i}^{-}$, and hence *v*_*i*_ = *w*_1−*i*_. Since *ϵ* < 0.5, we have *γ*_0_ > *γ*_1_; ${C}_{s}^{{v}_{0}}-{C}_{s}^{{v}_{1}}\ge 0$ by the definition of *H*^+^, which proves the inequality.

Having shown that the solution of maximal likelihood under mild conditions is, intuitively, that which has concordant expression at each SNP locus *s*, we now measure the probability of concordant expression at that SNP, and only phase when that probability is sufficiently high, in order to determine which SNPs can be phased with high accuracy. This probability of concordant expression can be immediately derived from Eq. (14). We assume a uniform error rate of *ϵ* for ease of notation, though is not required. Let CE(*R*_*g*,*s*_, *H*[*s*]) denote the event of concordant expression at *s*, then

15 $${\rm{P}}[{\rm{CE}}({R}_{g,s},H[s])| \beta ,\epsilon ]=\frac{{\rm{P}}[{R}_{g,s}| {H}^{+}[s],\beta ,\epsilon ]}{{\rm{P}}[{R}_{g,s}| {H}^{+}[s],\beta ,\epsilon ]+{\rm{P}}[{R}_{g,s}| {H}^{-}[s],\beta ,\epsilon ]}=\frac{1}{1+{\left(\frac{{\gamma }_{1}}{{\gamma }_{0}}\right)}^{| {C}_{s}^{0}-{C}_{s}^{1}| }}$$

Furthermore, given *N* reads, an expression bias *β*, and a constant error rate *ϵ*, we compute likelihood of concordant expression using the standard binomial distribution *B*(*N*, *γ*_0_) by equating successes in the binomial model to observations of the majority allele, expressed with bias *γ*_0_ (recall *γ*_*i*_ takes errors into account):

16 $${\rm{P}}[{\rm{CE}}| N,\beta ,\epsilon ]=\sum_{i = \lceil \frac{N+1}{2}\rceil }^{N} \left(\begin{array}{cc}N \\ i\end{array}\right){\gamma }_{0}^{i}{\gamma }_{1}^{N-i}\ge 1-{e}^{-N\frac{1}{2p}{(p-\frac{1}{2})}^{2}}$$

To obtain the bound on the right hand side, apply the Chernoff bound ${\rm{P}}[X {\,} < {\,} \left.(1-\lambda )\mu \right)] \, \le \, {e}^{-\frac{{\lambda }^{2}\mu }{2}}$, where *X* corresponds to the number of successes and *μ* = E[*X*] = *Nβ*. This bound shows that the probability of concordant expression increases exponentially with the coverage (*N*).

We remark for large *N*, the Binomial Distribution B(*n*, *β*) converges to the normal distribution ${\mathcal{N}}(N\beta ,N\beta (1-\beta ))$, and therefore this probability can always be easily computed.

### Likelihood of non-biased expression

Now that we have a method for determining the likelihood of concordant expression, we can require any SNP loci to have a sufficiently high probability of concordant expression in order for HapTree-X to attempt to phase that SNP. The likelihood of concordant expression is dependent on *β* however, which we may only estimate. We therefore also require that for any gene *g* to be phased by DASE (or, alternatively, particular SNP *s*), the DASE within the gene (at *s*) must be sufficiently unlikely to have been generated by uniform DHE (*β* = 0.5) (because in this case, we cannot use DASE-based methods to phase).

We compute an upper bound on this probability using a two-sided binomial test applied to total allele counts *m*, *M*, where

17 $$m=\sum_{s\in g}\min (C _{s}^{0} ,{C}_{s}^{1})\,{\text{and}}\,M=\sum_{s\in g}\max (C _{s}^{0} ,{C}_{s}^{1})$$

for the case of a gene *g*. For a single SNP *s*, we write

18 $$m=\min (C _{s}^{0} ,{C}_{s}^{1})\,{\text{and}}\,M=\max (C _{s}^{0} ,{C}_{s}^{1}).$$

The likelihood of at least *M* heads and at most *m* tails is computed below. Let *N* = *m* + *M*, then the upper bound based on the two-sided binomial test is

19 $$\sum_{i = 0}^{m}\left(\begin{array}{cc}N \\ i\end{array}\right){\frac{1}{2}}^{N}+\sum_{i = M}^{N}\left(\begin{array}{cc}N \\ i\end{array}\right){\frac{1}{2}}^{N}.$$

As mentioned above, the Binomial distribution *B*$(n,\frac{1}{2})$ converges to the normal distribution ${\mathcal{N}}(\frac{N}{2},\frac{N}{4})$, and therefore we may efficiently compute these likelihoods.

### Reporting summary

Further information on research design is available in the Nature Research Reporting Summary linked to this article.

## Supplementary information

Supplementary Information

Peer Review File

Reporting Summary

## Data Availability

The complete experimental pipeline, the relevant software, and the relevant data download links are available in the Jupyter Notebook format at http://haptreex.csail.mit.edu and https://github.com/0xTCG/haptreex/. The RNA-seq sequencing data for GM12878 (nucleus, cytosol and whole) and K562 cell lines are available through ENCODE project (track wgEncodeCshlLongRnaSeq; the exact accession IDs are listed in the Supplementary Note 2). 10× samples (NA12878 and NA24385) are available from 10× Genomics de novo Assembly collection (Supernova 2.0.0; https://www.10xgenomics.com/resources/datasets/). Whole exome data are available in BAM format through 1000 Genomes Phase 3 (ID: NA12878, version: 20121211). The GIAB RNA-seq data (NA12878, NA24143, NA24219, NA24385, and NA24631) are available for download at http://haptreex.csail.mit.edu/datasets. NA12878 WGS sample (BAM and VCF) is available through Illumina Platinum Genomes project (gs://genomics-public-data/platinum-genomes). The validation VCFs datasets are available through the Genome in the Bottle project (https://github.com/genome-in-a-bottle/giab_latest_release). GEUVADIS samples are available through 1000 Genomes project; the exact accession IDs are listed in the Supplementary Note 2. All relevant data supporting the key findings of this study are available within the article and its Supplementary Information files or from the corresponding author upon reasonable request.

## Code availability

The HapTree-X software is free and open source and is available at http://haptreex.csail.mit.edu.
