# Supplementary material for: Improved haplotype inference by exploiting long-range linking and allelic imbalance in RNA-seq datasets
Source: Nat Commun. 2020 Sep 16;11:4662. doi: 10.1038/s41467-020-18320-z (PMC7494856; doi:10.1038/s41467-020-18320-z)
Supplement: Supplementary file 1 — Supplementary Information [file 41467_2020_18320_MOESM1_ESM.pdf]

Improved haplotype inference by exploiting long-range linking and allelic imbalance in RNA-seq datasets

Supplementary Notes and Figures

Emily Berger, Deniz Yorukoglu, Lillian Zhang, Sarah Nyquist,
Alex K. Shalek, Manolis Kellis, Ibrahim Numanagić and Bonnie Berger

Contents

Supplementary Note 1: Theoretical Performance of DASE **2**

Supplementary Note 2: Experimental Setup **3**

2.1 GIAB RNA-seq sequencing . . . 4

Supplementary Note 3: Phasing and Multiple Isoforms **5**

3.1 Phasing in the Presence of Multiple Isoforms . . . 5

3.1.1 Definitions and Notation . . . 5

3.1.2 Matrix Representation . . . 6

3.1.3 Problem Statement . . . 6

3.1.4 Zonotope Representation . . . 7

3.1.5 Phasing Two SNPs Given Multiple Isoforms . . . 8

3.1.6 Modeling Noise . . . 9

3.1.7 Experimental Results . . . 10

Supplementary Figures **13**

Supplementary References **20**

Supplementary Note 1: Theoretical Performance of DASE

We demonstrate in **Methods** the differential haplotypic expression level of a gene, β, and its coverage determine likelihood of concordant expression. We show this relationship below for varying β and levels of coverage. While these functions are derived from an idealized model of the data, this relationship suggests that as the depth-coverage of a dataset increases, so does the likelihood of concordant expression, and hence the accuracy of HapTree-X. Supplementary Figure 1 displays the theoretical curves depicting the exponential growth of likelihood of concordant expression as a function of coverage and β, the differential haplotypic expression. We infer from this theoretical result that requiring a lower bound of DHE is beneficial for reliable DASE-based phasing given moderate coverage. Furthermore, Supplementary Table 1 shows minimum coverage required to obtain a probability of at least $1 - 10^{-\alpha}$ of concordant expression, given β.

As discussed in **Methods**, the exact solution of maximum likelihood (for any gene g; Equation (9) in the Methods) corresponds to that with concordant expression at all SNP loci within g. HapTree-X therefore uses a threshold λ (negative log-likelihood of concordant expression) which requires any SNP to be concordantly expressed with probability at least $1 - e^{-\lambda}$, in order to be phased. We run HapTree-X while varying this threshold λ; we compute the percentage of concordantly expressed SNPs and the total phased SNPs as we increase this threshold. As the threshold increases, HapTree-X demands any SNP to be phased to have a correspondingly high likelihood of concordant expression; as a result, the phasing accuracy of HapTree-X increases. The cost paid for this increase in accuracy is a decrease in the total number of SNPs phased, as seen in Supplementary Figure 2.

For the results reported, we used a threshold value of 20. In theory, this threshold value λ would produce a percentage of concordantly expressed SNPs equal to $1 - e^{-\lambda}$; however because of the structural noise commonly observed in aligned RNA-seq data due to false mapping, RNA-editing, as well complex alternative splicing events, we require a $\lambda' > \lambda$ to meet desired accuracy levels. Additionally, motivated by the above-mentioned discussion we require an estimated $\beta \geq 0.6$ for any gene to be phased using DASE, as it represents a reasonable compromise between the concordance probability and potential number of SNPs that are affected by DASE at that level (for a visualisation of β values please see Figure 1). Finally, we have several methods for managing alternative splicing events.

HapTree-X optionally can avoid all genes with alternative splicing, phase s, s' only if the set of isoforms containing s, s' are equal, or alternatively phase independent of isoforms but require s, s' to have coverage and DASE that are sufficiently similar. This third option was used in the experiments with RNA-seq datasets. The first two options result in higher accuracy for lower λ, but of course phase fewer SNPs.

Tables

$\beta \setminus \alpha$	**2**	**3**	**4**	**5**
0.85	9	15	21	27
0.80	13	21	31	41
0.75	19	33	49	63
0.70	31	55	79	105
0.65	57	101	147	193
0.60	133	235	339	447
0.55	539	951	1377	1811

Supplementary Table 1: Coverage needed to obtain likelihood $1 - 10^{-\alpha}$ of concordant expression given a differential haplotypic expression of β and an assumed opposite allele error rate of 2%.

Supplementary Note 2: Experimental Setup

We evaluate haplotype reconstruction performance of HapTree-X on diploid RNA-seq, whole-genome, whole-exome, and combined sequencing datasets from a human female individual with European ancestry (GM12878), and compare the results to several other sequence-based phasing methods. We repeat this evaluation of HapTree-X on diploid RNA-seq, whole-exome, and combined sequencing datasets on the five Genome in a Bottle (GIAB) samples: NA12878, NA24143, NA24149, NA24385, and NA24631, and extend it to 10X Genomics data for two individuals NA12878 and NA24385. We also ran HapTree-X on Platinum Genomes NA12878 WGS sample. Finally, we evaluated HapTree-X on the ENCODE cell line K562 (`wgEncodeCshlLongRnaSeqK562Nucleolus`). More details can be found in our experimental notebook at https://haptreex.csail.mit.edu.

GM12878 RNA-seq raw read datasets of a lymphoblastoid cell-line from NA12878 (GM12878) are obtained from ENCODE CSHL Long RNA-seq (`wgEncodeCshlLongRnaSeq`) track with average sequencing depth of 100 million mate-pairs (2x76bp), transcriptome fragments sequenced from the nucleus, cytosol and whole-cell with Poly-A$^+$ and Poly-A$^-$ profiling. Exact file IDs are available in Supplementary Table 2.

Thirty GEUVADIS samples from the 1000 Genomes project that were used to validate the clinical utility of HapTree-X are listed in Supplementary Table 2.

Sample	ID
Cytosol	`wgEncodeCshlLongRnaSeqGm12878CytosolLongnonpolya`
	`wgEncodeCshlLongRnaSeqGm12878CytosolPap`
	paired-end files with both replicates (Rep1 and Rep2) merged together
Nucleus	`wgEncodeCshlLongRnaSeqGm12878NucleusLongnonpolya`
	`wgEncodeCshlLongRnaSeqGm12878NucleusPap`
	paired-end files with both replicates (Rep1 and Rep2) merged together
Whole	`wgEncodeCshlLongRnaSeqGm12878CellLongnonpolya`
	`wgEncodeCshlLongRnaSeqGm12878CellPap`
	paired-end files with both replicates (Rep1 and Rep2) merged together
K562	`wgEncodeCshlLongRnaSeqK562NucleolusTotal`
	paired-end files of replicate #3

Part (1)

ERR188033	ERR188080	ERR188150	ERR188246
ERR188312	ERR188340	ERR188379	ERR188426
ERR188036	ERR188091	ERR188185	ERR188262
ERR188314	ERR188361	ERR188395	ERR188435
ERR188043	ERR188146	ERR188213	ERR188264
ERR188335	ERR188368	ERR188397	ERR188076
ERR188149	ERR188215	ERR188277	ERR188337
	ERR188371	ERR188418	

Part (2)

Supplementary Table 2: Accession IDs used in this study. Part (1) shows ENCODE CSHL Long RNA-seq IDs, while Part (2) contains relevant GEUVADIS IDs.

Pre-processing: All samples were aligned and genotyped by GATK Best Practices pipeline, and the resulting VCFs were fed to each phaser as an input to be phased. In all experiments, we aligned RNA-seq reads directly to the reference genome (hg19) rather than a reconstructed transcriptome based on a gene model, as a significant percentage of RNA-seq reads align better to the outside of the exonic regions rather than inside; forcing these reads to align to the transcriptome will likely cause incorrect alignments and hence add noise to genotyping and phasing algorithms. 10X Genomics datasets were aligned by EMA [1].

Validation: We assess the accuracy of phased haplotype blocks generated by HapTree-X, by comparing our phasing results to a high-quality GIAB phased VCFs as the ground truth. We restricted DASE-based phasing within HapTree-X only to the SNPs that are located within the same gene in the GENCODE gene annotation v19 (`wgEncodeGencodeCompV19`). K562 chronic myelogenous leukemia cell line RNA-seq data was validated with the recently-validated phasing ground truth dataset from ENCODE [2].

Tools: We compared our results to those of HapCUT v0.7, HapCUT2, and phASER v0.9. In the case of HapCUT and HapCUT2, to accommodate for long range splicing-junctions within RNA-seq read alignments, we defined maximum insert-size (maxIS) parameter to be longer than each chromosome's length. For HapCUT and phASER, we set minimum base quality (mbq, baseq) parameter to 0 to utilize maximum read contiguity information provided in the datasets (unless otherwise noted). We could not do the same for HapCUT2, so we used the default settings. All other parameters were set to be default.

2.1 GIAB RNA-seq sequencing

We repeated our RNA-seq and whole-exome experiments on the five Genome in a Bottle (GIAB) samples: NA12878, NA24143, NA24149, NA24385, and NA24631, and validated our results using high confidence variant calls from GIAB portal as ground truth, which corroborated our HapTree-X performance findings in the 1000 Genomes GM12878 individual.

RNA sequencing for each GIAB sample was done in-house. Cells were obtained from Coriell and cultured according to instructions (upright with unfiltered lids overnight then with filtered lids and antibiotics). One million cells from each strain were then flash frozen in lysis buffer (1%BME in RLT). Four replicate RNA extractions were done for each strain using the RNeasy Mini Kit (QIAGEN). RNA-Seq library prep was done with a modified version of the SMART-Seq2 protocol [3, 4]. Whole transcriptome amplification was performed using KAPA HiFi PCR Master mix (Kapa Biosystems KK2602) and IS PCR Primer (IDT). Size selection was done using Ampure DNA SPRI beads at 0.6x and 0.7x concentrations. Concentration was quantified using a Qubit. Sequencing libraries were prepared using the Nextera XT DNA tagmentation kit (Illumina FC-131-1096) with 250 pg input for each sample according to the manufacturer's instructions, and Ampure DNA SPRI bead size selection at 0.6x and 0.8x was done. Paired end sequencing was done using Illumina 300 Cycle NextSeq500/550v2 kit with loading density at 2.2 pM.

Supplementary Note 3: Phasing and Multiple Isoforms

3.1 Phasing in the Presence of Multiple Isoforms

We have seen, with HapTree-X, that it is possible to perform phasing based on differential haplotypic expression (DHE) by making use of differential allele specific expression (DASE). In the case of HapTree-X, we restricted phasing depending on the structure of the isoforms of a gene; phasing in the general case of multiple isoforms is much more complicated. We discuss some cases in which phasing when there are multiple isoforms can be achieved.

Phasing based on DHE and DASE is immediately made more complex by the existence of multiple isoforms. This is primarily because the rates of differential haplotypic expression are independent across isoforms, and furthermore, each isoform is expressed at a different frequency. Fortunately, methods for determining the relative frequencies of expression of isoforms, or *quantification*, have been studied in [5, 6]. We therefore will assume that these relative frequencies are known.

Not only are isoforms expressed at different frequencies, but the two haplotypes of any isoform may be differentially expressed as well; it is this differential expression that we wish to use for haplotype reconstruction. Unfortunately, these DHE rates are not known and we will see that depending on the situation they may or may not be able to be determined. Furthermore, we will see that knowing the differential expressions may not always be enough to determine the haplotypes, and vice versa. We explore this problem here.

3.1.1 Definitions and Notation

Recall the goal of phasing is to recover the unknown haplotypes, $H = (H_0, H_1)$, which contain the sequence of variant alleles inherited from each parent of the individual. From now on, we will restrict ourselves to one gene, G, with isoforms $\{I_1, ..., I_k\}$. Let S_i denote the set of heterozygous SNPs that are present within the exons of the isoform I_i; we let $S = \cup_i S_i$. For any $s \in S$, we let $H_0[s], H_1[s]$ denote the allele present at s on the haplotypes respectively.

Suppose $|S| = n$ and $S = \{s_1, ..., s_n\}$, we can define the *Isoform Matrix* M (an incidence matrix), whose rows correspond to the isoforms I_i and columns correspond to the set of SNPs S; that is $M = \{m_{i,j}\}$ where

$$m_{i,j} = \begin{cases} 1 & \text{if } s_j \in S_i \\ 0 & \text{if } s_j \notin S_i \end{cases}$$

We assume that the quantification of the isoforms is known; that is each isoform I_i is expressed at a rate proportional to α_i

For each SNP s_j, we define the relative SNP expression level as A_j, where

$$A_j = \sum_{i : s_j \in I_i} \alpha_i.$$

Let β_i^M and β_i^P (with $\beta_i^M + \beta_i^P = 1$) denote the expressions of the maternal and paternal haplotypes respectively for isoform I_i; let $\delta_i = (\beta_i^M - \beta_i^P)/2$.

We assume that M has all distinct rows, that is: isoforms covering identical sets of SNPs are considered to be the same. If this is not the case, say $I'_1, ..., I'_{k'}$ are all identical with quantifications $q'_1, ..., q'_{k'}$ and differential expressions $\delta'_1, ..., \delta'_{k'}$, then we may represent these isoforms as one isoform I' covering the same set of SNPs with quantification $q' = \sum q'_i$ and differential expression $\delta' = \sum q'_i \delta'_i$.

Finally, we let

$$\sigma_j = \begin{cases} 1 & \text{if } H_0[s_j] = 0 \\ -1 & \text{if } H_0[s_j] = 1 \end{cases}$$

Suppose we are given, for each SNP s_j, the proportion of alleles expressed that are the reference allele (0) and alternative allele (1); we denote these as v_j^0 and v_j^1, respectively. In our ideal model of the world, all isoforms are known exactly, each isoform is expressed infinitely (in proportion to its quantification), no read

covers more than one SNP, and differential expression is exactly constant across an isoform. In this case, the following should hold:

$$v_j^0 = \frac{1}{A_j} \sum_{i=1}^{k} m_{i,j} \alpha_i \left(\frac{1}{2} + \sigma_j \delta_i \right) \tag{1}$$

$$v_j^1 = \frac{1}{A_j} \sum_{i=1}^{k} m_{i,j} \alpha_i \left(\frac{1}{2} - \sigma_j \delta_i \right) \tag{2}$$

3.1.2 Matrix Representation

It will be useful to formulate these relations in terms of matrices. Let $Q = \{q_{i,j}\}$ be the $k \times k$ diagonal matrix with entries corresponding to the quantifications levels, that is: $q_{i,i} = \alpha_i$. Let $A = \{a_{i,j}\}$ be the $n \times n$ diagonal matrix containing the relative SNP expression levels A_j, that is $a_{j,j} = A_j$. We can then define the *Weighted Isoform Matrix*, $M' = \{m'_{i,j}\}$, where

$$M' = QMA^{-1}.$$

By definition $m'_{i,j} = \frac{\alpha_i m_{i,j}}{A_j}$.

The relations described in 1 and 2 can be represented equivalently as matrix relations as well. To do so, we define $\delta = (\delta_1, ..., \delta_k)$ (with $-\frac{1}{2} \leq \delta_i \leq \frac{1}{2}$) and let $S = \{s_{i,j}\}$ be the $n \times n$ diagonal matrix containing the σ_j, with $s_{j,j} = \sigma_j$. Finally, let $v^0 = (v_1^0, v_2^0, ..., v_n^0)$ (and let v^1 be defined analogously), then 1 may be expressed as

$$v^0 = f_k M' + \delta M' S$$
$$v^1 = f_k M' - \delta M' S$$

where $f_k = \left(\frac{1}{2}, \frac{1}{2}, ..., \frac{1}{2}\right)$ and is of length k. Since the columns of M' each sum to one, $f_n = f_k M'$.

Let $v = (v^0, v^1)$, since $v^0 + v^1 = (1, ..., 1)$, without loss of generality will restrict ourselves to v^0. Let

$$z = v^0 - f_n = \delta M' S \tag{3}$$

$$z' = (v^0 - f_n) S^{-1} = \delta M'. \tag{4}$$

S is invertible as $\sigma_j = \pm 1$; indeed $S = S^{-1}$.

We will see that being able to phase with multiple isoforms is equivalent to the existence of unique (up to sign) S such that there exists $|\delta_i| \leq \frac{1}{2}$ where S, δ satisfies 3.

3.1.3 Problem Statement

We assume an idealized model of the world, where all isoforms of a gene are perfectly known, as are their quantifications. Furthermore, each isoform is expressed infinitely (in proportion to its known quantification), no read covers more than one SNP, and differential expression is exactly constant across an isoform (though unknown). Given total proportions of reference and alternative alleles being expressed from all isoforms at each SNP locus, we can ask for how many possible pairs of haplotypes, given the known isoforms and their quantifications, it is feasible for us to have observed these allele proportions. In the case where there is a unique pair of haplotypes, we say the haplotypes can be recovered and there exists a unique haplotype solution.

In our notation from the previous section, we assume that the differential expressions δ and haplotypes S are fixed but unknown. We are given the isoforms M, their quantifications Q (and from M and Q we may deduce A and therefore M'). Finally we observe v, and equivalently z. The existence of a unique set of haplotypes is equivalent to there existing a unique (up to sign) S such that there exists $|\delta| \leq \frac{1}{2}$ where $z = \delta M' S$.

Definition 1. *For a given M' and z, S is a feasible haplotype solution if there exists $|\delta| \leq \frac{1}{2}$ such that $z = \delta M' S$.*

Definition 2. *For a given M' and z, the haplotypes may be recovered if there exists a unique (up to sign) feasible solution $\pm S$.*

3.1.4 Zonotope Representation

We are fortunate to be able to borrow from the rich theory of polytopes, zonotopes, and hyperplane arrangements to describe the set of observations z for which a particular haplotype solution S is feasible. We will state several facts about zonotopes without proof; we refer the reader to [7].

A zonotope may be defined as the Minkowski sum of a set of line segments beginning at the origin. Given a set of points $X \subset V$ (spanning V), the zonotope $Z(X) \subset V$ may be written as

$$Z(X) = \left\{ \sum_{x \in X} t_x x \mid 0 \leq t_x \leq 1 \right\}.$$

We can shift $Z(X)$, to $Z'(X)$, so that it is centered at the origin, by subtracting the point $\sum_{x \in X} \frac{1}{2} x$ from all points $z \in Z(X)$; equivalently we can write

$$Z'(X) = \left\{ \sum_{x \in X} t_x x \mid -\frac{1}{2} \leq t_x \leq \frac{1}{2} \right\}.$$

It is known (but non-trivial to show) that $Z(X)$ (or any polytope) may be written as the intersection over a set of half-spaces each defined by a hyperplane. For the case of zonotopes, there is a nice way to define these hyperplanes, described in Proposition 2.43 of [7]. To do so, let F denote the set of subsets of X that span a codimension one subspace of V. For each $f \in F$, let u_f be a linear equation such that $\langle u_f, v \rangle = 0$ defines the subspace spanned by f. Finally, define μ_f^- and μ_f^+ to be the minimum and maximum values attained by f over $Z(X)$, respectively. With these definitions in mind, Proposition 2.43 of [7] states that $Z(X)$ may be written as

$$\bigcap_{f \in F} \left\{ v \in V \mid \mu_f^- \leq \langle u_f \mid v \rangle \leq \mu_f^+ \right\}. \quad (5)$$

Let $B^- := \{x \in X \langle u_f \mid x \rangle < 0\}$ and $B^+ := \{x \in X \langle u_f \mid x \rangle > 0\}$. In the case of $Z(X)$, μ_f^- and μ_f^+ are the sums of the values of u_f at x, for x in B^- and B^+, respectively.

$$\mu_f^- := \sum_{b \in B^-} \langle u_f, b \rangle; \quad \mu_f^+ := \sum_{b \in B^+} \langle u_f, b \rangle.$$

In the case of the shifted zonotope $Z'(X)$, u_f takes the following minimum and maximum values on $Z'(X)$:

$$\mu_f^- := \sum_{b \in B^-} \frac{1}{2} \langle u_f, b \rangle + \sum_{b \in B^+} -\frac{1}{2} \langle u_f, b \rangle; \quad \mu_f^+ := \sum_{b \in B^+} \frac{1}{2} \langle u_f, b \rangle + \sum_{b \in B^-} -\frac{1}{2} \langle u_f, b \rangle.$$

We will use this half-space representation to describe the observations z for which a particular haplotype solution S if feasible.

For any fixed S, the set of z satisfying 3 are those points in the shifted zonotope $Z'(X)$, where X is the set of rows of the matrix $M'S$; we refer to this zonotope as $Z'_S(M')$. Recall S is the diagonal matrix, with entries $\sigma_j = \pm 1$, representing the haplotypes. Without loss of generality, we consider only $Z'(M') = Z'_I(M')$, where I is the identity matrix. We may restrict to this case because any $Z'_S(M)$ is the reflection of $Z'(M)$ through the set of coordinates j such that $\sigma_j = -1$.

Following 5, to determine $Z'(M')$, we must compute the linear equations defining the hyperplanes spanned by subsets of rows M' and their minimum and maximum values on $Z'(M')$. To understand the codimension one subspaces spanned by the row vectors of M' it is enough to understand those spanned by the row vectors of M because $M' = QMA^{-1}$, where both Q and A are diagonal. If a subset of rows of M span the subspace defined by

$$\sum_{j=1}^{n} \gamma_j x_j = 0,$$

then the corresponding rows in M' span the subspace defined by

$$\sum_{j=1}^{n} \gamma_j A_j x_j = 0.$$

For fixed n, the most general isoform matrix (which we will denote as $\hat{M}(n)$) is the collection of all non-empty binary strings. Determining the linear equations defining all codimension one subspaces spanned by the row vectors of $\hat{M}(n)$ is sufficient for understanding the hyperplane description of any $Z'(M')$, provided M' has at most n columns.

In the following section, we define the hyperplane descriptions of $Z'(M')$ and their corresponding minima and maxima for the case when $n = 2$. The cases of $n = 3$ and $n = 4$ are included at the end of this section.

3.1.5 Phasing Two SNPs Given Multiple Isoforms

In the case where a gene G covers exactly two SNPs, we may write down simple necessary and sufficient conditions for the existence of a unique haplotype solution S given z, Q, and M. Recall that we may assume that M has no duplicate rows (that is, isoforms covering identical sets of SNPs are considered to be the same.) Therefore, without loss of generality, we can write:

$$Q = \begin{pmatrix} \alpha_1 & 0 & 0 \\ 0 & \alpha_2 & 0 \\ 0 & 0 & \alpha_3 \end{pmatrix} \qquad M = \begin{pmatrix} 1 & 0 \\ 0 & 1 \\ 1 & 1 \end{pmatrix} \qquad A = \begin{pmatrix} \alpha_1 + \alpha_3 & 0 \\ 0 & \alpha_1 + \alpha_3 \end{pmatrix}$$

$$M' = QMA = \begin{pmatrix} \frac{\alpha_1}{\alpha_1+\alpha_3} & 0 \\ 0 & \frac{\alpha_2}{\alpha_1+\alpha_3} \\ \frac{\alpha_3}{\alpha_1+\alpha_3} & \frac{\alpha_2}{\alpha_2+\alpha_3} \end{pmatrix}.$$

We investigate what kind of solution pairs δ, S satisfy $zS = \delta M'$ with $\frac{-1}{2} \leq \delta_i \leq \frac{1}{2}$ and $S = \left(\begin{smallmatrix} \sigma_1 & 0 \\ 0 & \sigma_2 \end{smallmatrix}\right)$, with $\sigma_j \in \{\pm 1\}$. The case $S = \pm \left(\begin{smallmatrix} 1 & 0 \\ 0 & 1 \end{smallmatrix}\right)$ corresponds to the case in which the two SNPs are *phased in parallel* (the reference allele occurs at both SNP sites on one haplotype and the alternative allele at both SNP sites on the other.) The case $S = \pm \left(\begin{smallmatrix} -1 & 0 \\ 0 & 1 \end{smallmatrix}\right)$ corresponds to the SNPs having *switched phase* (one haplotype has a reference allele followed by alternative allele and the other haplotype an alternative allele followed by a reference allele.) Note that if S, δ is a solution, then $-S, -\delta$ is also a solution and corresponds to relabelling the haplotypes (switching the maternal and paternal haplotypes), and we therefore restrict ourselves to the cases say $S_p = \left(\begin{smallmatrix} 1 & 0 \\ 0 & 1 \end{smallmatrix}\right)$ and $S_s = \left(\begin{smallmatrix} -1 & 0 \\ 0 & 1 \end{smallmatrix}\right)$.

It is possible to determine the phase of the gene G if there do not exist both forms of solution pairs S_p, δ and S_s, δ; having solutions of both forms imply z can be explained by either parallel or switched phasing, and therefore the phasing is ambiguous. We derive conditions on z which determine when z is such that it is feasible for G to be parallel phased; equivalently when there exist solution pairs S_p, δ satisfying $zS_p = \delta M'$ and $-\frac{1}{2} \leq \delta_i \leq \frac{1}{2}$. We also derive the corresponding conditions for the case of switched phase. The phasing of G is ambiguous when z satisfies both sets of conditions.

From the viewpoint of section **Zonotope Representation**, we wish to describe $Z'_{S_p}(M')$, its reflection across the $y-$axis, $Z'_{S_s}(M')$, and their intersection; any point z in the intersection $Z'_{S_p}(M') \cap Z'_{S_s}(M')$ implies the phasing of G is ambiguous. In the discussion that follows, we think of $Z'(M')$ as the projection of the cube Δ defined by $-\frac{1}{2} \leq \delta_i \leq \frac{1}{2}$ by multiplication on the right by M' (Supplementary Figure 3).

To determine conditions for when z can be explained by parallel phasing, we derive equations for the hyperplane description of the zonotope $Z'_{S_p}(M')$ in terms of z and Q.

Proposition 1. *The following conditions are necessary and sufficient for the existence of $\delta \in \Delta$ such that $(x, y) = z = \delta M'$, where Δ is the cube defined by $|\delta_i| \leq \frac{1}{2}$:*

$C_y : |y| \leq \frac{1}{2}$,

$C_x : |x| \leq \frac{1}{2}$, *and*

$C^-_{xy} : |(\alpha_2 + \alpha_3)y - (\alpha_1 + \alpha_3)x| \leq \frac{\alpha_1+\alpha_2}{2}$.

Proof. From the discussion in section **Zonotope Represenation**, we begin by enumerating the linearly independent subsets of M' who span a codimension 1 subspace, trivially these are the rows r_1, r_2, r_3 of M'. Equating (x, y) to $\delta M'$ implies the following equations:

$$(\alpha_1 + \alpha_3)x = \alpha_1\delta_1 + \alpha_3\delta_3 \tag{6}$$

$$(\alpha_2 + \alpha_3)y = \alpha_2\delta_2 + \alpha_3\delta_3 \tag{7}$$

$$(\alpha_2 + \alpha_3)y - (\alpha_1 + \alpha_3)x = \alpha_2\delta_2 - \alpha_1\delta_1. \tag{8}$$

In general, the coefficient attached to δ_i on the RHS is what the row r_i evaluates to under the LHS. Each δ_i is missing from one these equations, and these equations are therefore sufficient for defining the zonotope as an intersection of half-spaces. To determine those half-spaces, we follow **Zonotope Represenation** and minimize and maximize the RHS of each equation, implying the bounds in the statement of Proposition 1.

□

Proposition 2. *The following conditions are necessary and sufficient for the existence of $\delta \in \Delta$ such that $(x, y) = z = \delta M' \left(\begin{smallmatrix}-1 & 0\\ 0 & 1\end{smallmatrix}\right)$, where Δ is the cube defined by $|\delta_i| \leq \frac{1}{2}$:*

$C_y : |y| \leq \frac{1}{2}$.

$C_x : |x| \leq \frac{1}{2}$ *and*

$C^+_{xy} : |(\alpha_2 + \alpha_3)y + (\alpha_1 + \alpha_3)x| \leq \frac{\alpha_1+\alpha_2}{2}$.

This statement follows from Proposition 1 by a reflecting across the y-axis.

By combining Propositions 1 and 2 we can determine when the phasing of G is ambiguous; that is, when both C^-_{xy} and C^+_{xy} hold.

We include Supplementary Figure 4 depicting the zonotopes $Z'_{S_p}(M')$ and $Z'_{S_s}(M')$ as determined by 1 and 2. As intuition suggests, the larger α_3 is to relative to α_1, α_2, the smaller the set of z with ambiguous phasing ($Z'_{S_p}(M') \cap Z'_{S_s}(M')$). This intuition can be explained by the idea that I_3 is the only isoform with information about the true phase.

3.1.6 Modeling Noise

The model described in the preceding section assumed a theoretical set up where coverages of isoforms are sent to infinity in proportion to their quantifications, and the differential expression across an isoform is exactly constant. In reality, we do not have infinite coverage. Furthermore, we can have variation of coverage relative to the 'known' quantifications (of an entire isoform or just a SNP) and variation of differential expression within an isoform. These variations can be caused by both random and structural noise. Modeling all of these variations at once is equivalent to finding solutions δ, S to the equations

$$v^0_j = \frac{1}{A'_j}\sum_{i=1}^{k} m_{i,j}(\alpha_i + \epsilon_{\alpha,i,j})\left(\frac{1}{2} + \sigma_j(\delta_i + \epsilon_{\delta,i,j})\right) \tag{9}$$

$$v^1_j = \frac{1}{A'_j}\sum_{i=1}^{k} m_{i,j}(\alpha_i + \epsilon_{\alpha,i,j})\left(\frac{1}{2} - \sigma_j(\delta_i + \epsilon_{\delta,i,j})\right) \tag{10}$$

where $A'_j = \sum_{i=1}^{k} m_{i,j}(\alpha_i + \epsilon_{\alpha,i,j})$, such that $|\delta_i| \leq \frac{1}{2}$ and $\sigma_j = \pm 1$, the $\epsilon_\alpha, \epsilon_\delta$ are bounded in some way, and possibly $|\delta_i + \epsilon_{\delta,i,j}| \leq \frac{1}{2}$ as well. Even for fixed S, this problem is non-linear since the errors and δ are unknown. Instead, we discuss the simpler case of modeling variation of differential expression across an isoform.

Assuming fixed and known quantifications, we can model variation at the SNP level of differential expression. Without noise, differential expression ought to be the same for all SNPs across an isoform. We introduce noise limits $\varepsilon = \{\varepsilon_{i,j}\}$ to account for this variation. Given an observation v^0, v^1, we say S is a feasible haplotype solution if there exists $|\delta| \leq \frac{1}{2}$ and $\epsilon \leq \varepsilon$ (where $\{\epsilon_{\delta,i,j}\}$) such that the following hold:

$$v^0_j = \frac{1}{A_j}\sum_{i=1}^{k} m_{i,j}\alpha_i\left(\frac{1}{2} + \sigma_j(\delta_i + \epsilon_{\delta,i,j})\right)$$

$$v_j^1 = \frac{1}{A_j} \sum_{i=1}^{k} m_{i,j} \alpha_i \left(\frac{1}{2} - \sigma_j (\delta_i + \epsilon_{\delta,i,j}) \right)$$

$$z_j = v_j^0 - f_n = \frac{\sigma_j}{A_j} \sum_{i=1}^{k} m_{i,j} \alpha_i (\delta_i + \epsilon_{\delta,i,j}) \tag{11}$$

Recall $f_k = (\frac{1}{2}, \frac{1}{2}, ..., \frac{1}{2})$ and is of length k and that the column sums of M' are one.

Furthermore, we may wish to bound $|\delta_i + \epsilon_{\delta,i,j}| \leq \frac{1}{2}$ since those limits correspond to only one haplotype being expressed.

Let $\hat{\epsilon_j}, \hat{\varepsilon_j}$, denote the weighted average of errors and their upper bounds respectively at each SNP, that is

$$\hat{\epsilon_j} = \frac{1}{A_j} \sum_{i=1}^{k} m_{i,j} \alpha_i \epsilon_{\delta,i,j}$$

$$\hat{\varepsilon_j} = \frac{1}{A_j} \sum_{i=1}^{k} m_{i,j} \alpha_i \varepsilon_{i,j}.$$

Setting $\hat{\epsilon} = (\hat{\epsilon_1}, ..., \hat{\epsilon_n})$ and $\hat{\varepsilon} = (\hat{\varepsilon_1}, ..., \hat{\varepsilon_n})$, we may write

$$z = v^0 - f_k = \delta M' S + \hat{\epsilon} S.$$

and therefore

$$|z - \delta M' S| \leq \hat{\varepsilon}. \tag{12}$$

If we impose $|\delta_i + \epsilon_{\delta,i,j}| \leq \frac{1}{2}$, then $|z| \leq \frac{1}{2}$ as well.

For fixed S and given z, if we insist that $|\delta_i + \epsilon_{\delta,i,j}| \leq \frac{1}{2}$, then the existence of $|\delta| \leq \frac{1}{2}$ satisfying 12 does not imply the existence of $|\delta| \leq \frac{1}{2}, \epsilon \leq \varepsilon$ satisfying 11. If $|z| \leq \frac{1}{2}$ as it will be in practice, then there will exist a solution where the weighted average over i of $|\delta_i + \epsilon_{\delta,i,j}|$ is less than a half for each j.

3.1.7 Experimental Results

We wish to get a sense for whether or not phasing when multiple isoforms are present is possible with the current state of RNA-seq data. To do so, we see how accurate both our idealized and error based models are when implemented on RNA-seq data. We use RNA-seq raw read datasets of GM12878 obtained from ENCODE CSHL Long RNA-seq (wgEncodeCshlLongRnaSeq) track with average sequencing depth of 100 million mate-pairs (2x76bp), and transcriptome fragments sequenced from three different subcellular compartments: whole cell, cytosol, and nucleus. Also obtained from ENCODE CSHL Long RNA-seq are the transcript quantifications based on GENCODE gene annotation v7. We take as ground truth trio-phased SNPs of NA12878 from 1000 Genomes Project.

Based on the gene model mentioned above, we find all *isoform covered* SNP pairs $(s1, s2)$ (that is, those which occur together in an isoform with non-zero quantification; from here on, when we say isoform, we mean an isoform with non-zero quantification.) We can ask what the coverage at $(s1, s2)$ alone says about the feasible phasing solutions of the pair. This problem can be reduced to the setup in section 3.1.5 by letting α_1, α_2 and α_3 equal the sum of quantifications of all isoforms covering only $s1$, only $s2$, and both $s1$ and $s2$ respectively. Suppose the pair has coverage $[A_1, B_1]$ and $[A_2, B_2]$ where A_i, B_i denote the number of reads containing the reference allele at s_i and the alternative allele at s_i respectively. In our previous notation, we can write $(x, y) = (\frac{A_1}{A_1+B_1} - \frac{1}{2}, \frac{A_2}{A_2+B_2} - \frac{1}{2})$ and check when either $C^-_{x,y}$ (1) holds, which would mean parallel phasing is feasible, and when $C^+_{x,y}$ (2) holds, implying switched phasing is feasible.

$$C^-_{xy}: \ |(\alpha_2 + \alpha_3)y - (\alpha_1 + \alpha_3)x| \leq \frac{\alpha_1 + \alpha_2}{2}$$

$$C^+_{xy}: \ |(\alpha_2 + \alpha_3)y + (\alpha_1 + \alpha_3)x| \leq \frac{\alpha_1 + \alpha_2}{2}$$

In this context, it is possible to have two, one, or zero feasible solutions. In order for phasing with multiple isoforms to be effective, we ideally would like there to exist a unique feasible solution a high fraction of the time, and furthermore for that solution to be the true phasing of the pair. In the table below we count the number of pairs with two, one, and zero feasible solutions. We also report how often the unique feasible solution is accurate (AUFS), by which we mean agrees with the phasing provided in the gold-standard platinum VCF. We also report the same stats in the case where we condition on the coverage being at least 15 for both SNPs; somewhat surprisingly this does not affect AUFS significantly.

To incorporate error into the model, we follow the formulation in 12 and ask, for fixed ε, when there exists a feasible solution to

$$|z' - \delta M' S| \leq \varepsilon. \tag{13}$$

Equivalently, for which z' does there exist z with $|z' - z| \leq \varepsilon$ satisfying:

$$z = \delta M' S.$$

Let $z' = (x', y')$, it follows from 1 that the set of z' satisfying 13 are those which satisfy:

$$C_{xy}^{-}(\varepsilon): \; |(\alpha_2 + \alpha_3)y' - (\alpha_1 + \alpha_3)x'| \leq \frac{\alpha_1 + \alpha_2}{2} + (\alpha_1 + \alpha_2 + 2\alpha_3)\varepsilon$$

$$C_{xy}^{+}(\varepsilon): \; |(\alpha_2 + \alpha_3)y' + (\alpha_1 + \alpha_3)x'| \leq \frac{\alpha_1 + \alpha_2}{2} + (\alpha_1 + \alpha_2 + 2\alpha_3)\varepsilon$$

for $S = \pm \left(\begin{smallmatrix} 1 & 0 \\ 0 & 1 \end{smallmatrix}\right)$ and $\pm \left(\begin{smallmatrix} -1 & 0 \\ 0 & 1 \end{smallmatrix}\right)$ respectively. Note that z' will always satisfy C_x and C_y by definition.

For any $z' = (x', y')$, we can ask how much error is required in order for some S to be feasible; we let $f_p(x', y')$ and $f_s(x', y')$ denote such an error bound for parallel phased and switched phase solutions respectively:

$$f_p(x', y') = \frac{|(\alpha_2 + \alpha_3)y' - (\alpha_1 + \alpha_3)x'| - \frac{\alpha_1 + \alpha_2}{2}}{(\alpha_1 + \alpha_2 + 2\alpha_3)} \tag{14}$$

$$f_s(x', y') = \frac{|(\alpha_2 + \alpha_3)y' + (\alpha_1 + \alpha_3)x'| - \frac{\alpha_1 + \alpha_2}{2}}{(\alpha_1 + \alpha_2 + 2\alpha_3)}. \tag{15}$$

We can ask at various error levels, how many isoform covered SNP pairs have two, one, and zero feasible solutions (Supplementary Figure 5a). For the SNP pairs admitting only one feasible solution at a given allowed error bound, we compute the proportion of accurately phased SNP pairs (AUFS). As these curves do not vary significantly across Poly-A profile or replicate, we report these results for just Poly-A- replicate 2.

Generalizing one step further, we consider phasing a pair of SNPs when one phasing solution requires $\epsilon_m \leq \varepsilon_m$ error in order to be feasible and the other requires $\epsilon_M \geq \varepsilon_M$ error in order to be feasible. Using the notation introduced 14 and 15, we phase a SNP pair when either $f_p(x', y') \leq \varepsilon_m$ and $f_s(x', y') \geq \varepsilon_M$ or $f_s(x', y') \leq \varepsilon_m$ and $f_p(x', y') \geq \varepsilon_M$. When these conditions are satisfied, we compare the solution with lower error to the true phase. We refer to the upper bound ε_m as allowable min-error and the lower bound ε_M as required max-error. By doing so, we attempt to capture the intuition that given one solution which appears to be very good, the worse the alternate solution is, the higher our confidence the good solution is the true solution.

Below we plot how many SNP pairs can be phased for various $\varepsilon_m, \varepsilon_M$ (Supplementary Figure 7a) and how often those phasing solutions are accurate (Supplementary Figure 7b). Below, each curve corresponds to some $\varepsilon_m \in [0, .02, .05, .1]$. The enlarged points on the curves correspond to $\varepsilon_m = \varepsilon_M$; those points occur in Supplementary Figure 5b. In Supplementary Figure 6, for $\varepsilon_m = .05$, we plot the proportion of SNPs phased and phasing accuracy curves jointly. In all cases, we require coverage of at least 15 for all SNPs. As intuition suggests, accuracy appears to be approximately monotonically increasingly, and percentage of SNPs phased approximately monotonically decreasing, differing from the results presented in Supplementary Figure 5b.

Intuition suggests that with higher coverage, accuracy ought to increase; unfortunately requiring higher coverage limits how often we are able to phase. We fix $\varepsilon_m = .05$ and $\varepsilon_M = .15$ and for varying required coverage, we report what proportion of SNP pairs have the required amount of coverage , how accurately we phase when the coverage and error thresholds are satisfied (Supplementary Figure 8), and of SNP pairs

satisfying the coverage thresholds, what proportion satisfy the error thresholds and thus we choose to phase (Supplementary Figure 9).

We find that attempting to phase SNP pairs in the presence of multiple isoforms without taking into account error leads to low accuracy in phasing results, and furthermore for all replicates and both Poly-A profiles, between 55–60% of SNP pairs have no feasible solutions (Supplementary Table 3 and Supplementary Table 4).

If we incorporate allowable error, the percentage of SNP pairs with a unique feasible solution is maximized around an allowable error $\varepsilon = 0.05$. As we increase the allowable error, the accuracy rate of phasing increases, but the percentage of SNP pairs with two feasible solutions with goes to 100% (Supplementary Figure 5a), as it must, and therefore the percentage with a unique feasible solution goes to zero (Supplementary Figure 5b).

When we allow two dimensional error thresholds, that is, when in order to phase we require one solution be very close to 'perfect' (low required error) and the other to be very far from it (high required error), we see (unsurprisingly) for fixed allowable min-error, increasing required max-error increases accuracy while decreasing the proportion of SNPs phased (Supplementary Figure 6 and Supplementary Figure 7). Additionally, we see for fixed ε_m and ε_M, increasing coverage increases accuracy. In this context, for a fixed pair of error thresholds, the total number of SNP pairs able to be phased decreases as required coverage increases, though the proportion of SNP pairs (out of SNP pairs satisfying the lower bound of required coverage) which satisfy the error thresholds is noisy, but relatively flat when varying coverage.

At this time, it seems that certain pairs of SNPs can be accurately phased in the presence of multiple isoforms: those which satisfy various error and coverage thresholds. It may be useful to incorporate this sort of model into the general HapTree-X framework. To phase an entire gene of (possibly more than two) SNPs simultaneously, we can employ linear programming for each potential haplotype solution S for the gene to determine whether there exists $z = \delta M'S$ with $|\delta| \leq \frac{1}{2}$, or more generally if there exists a solution based on the error model for non-uniform differential expression in subsubsection 3.1.6. This approach unfortunately involves enumerating 2^{n-1} haplotypes, where n is the number of SNPs in the proposed gene, though is certainly feasible for some small $n > 2$ (and likely most genes).

Tables

**Results**	**PolyA- Rep 1**			**PolyA- Rep 2**		
# Feasible Solutions	2	1	0	2	1	0
# SNP Pairs	11,695	7,066	26,850	11,928	7,477	27,174
% SNP Pairs	25.64	15.49	58.87	25.81	16.05	58.34
**AUFS Rate**	0.6514			0.6580		

**Results**	**PolyA+ Rep 1**			**PolyA+ Rep 2**		
# Feasible Solutions	2	1	0	2	1	0
# SNP Pairs	15,618	8,714	34,682	14,778	8,473	33,561
% SNP Pairs	26.46	14.77	58.77	26.01	14.91	59.07
**AUFS Rate**	0.7592			0.7285		

Supplementary Table 3: Counts and percentage of number of isoform covered SNP pairs with two, one, or zero feasible solutions; accuracy of unique feasible solution (AUFS). No coverage requirement.

Results	PolyA- Rep 1			PolyA- Rep 2		
# Feasible Solutions	2	1	0	2	1	0
# SNP Pairs	3,891	1,062	6,360	4,019	963	6,189
% SNP Pairs	34.39	09.39	56.22	35.98	08.62	55.40
**AUFS Rate**	0.6271			0.6449		

Results	PolyA+ Rep 1			PolyA+ Rep 2		
# Feasible Solutions	2	1	0	2	1	0
# SNP Pairs	10,001	3,086	18,011	9,504	2,881	17,682
% SNP Pairs	32.16	09.92	57.92	31.61	09.58	58.81
**AUFS Rate**	0.7955			0.7372		

Supplementary Table 4: Counts and percentage of number of isoform covered SNP pairs with two, one, or zero feasible solutions; accuracy of unique feasible solution (AUFS). Coverage at least 15 for all SNPs.

Supplementary Figures

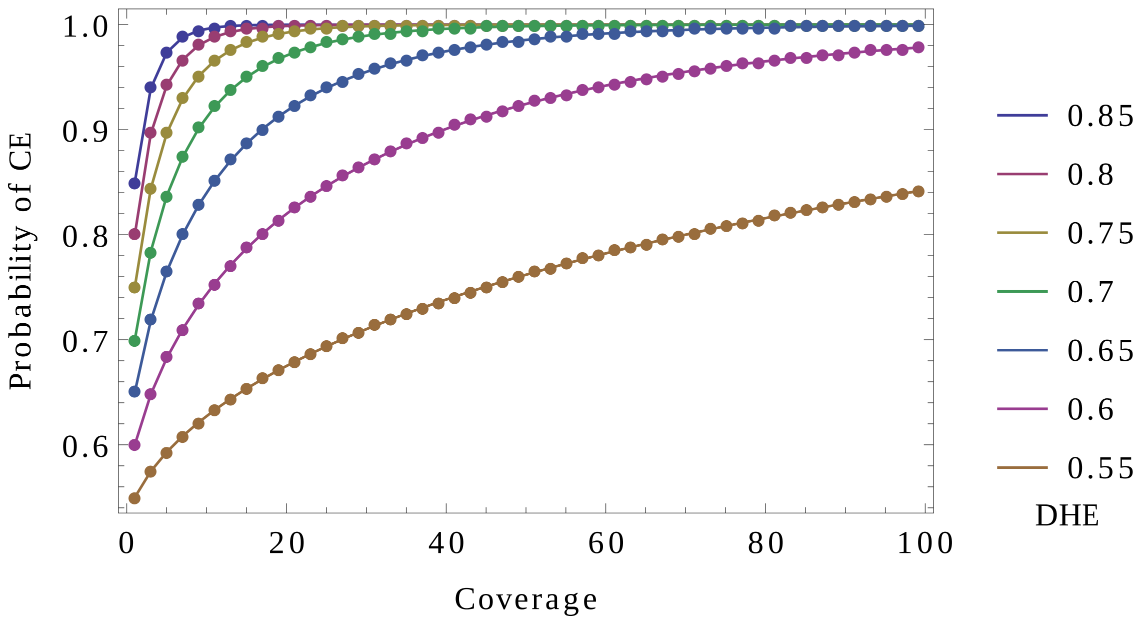

Supplementary Figure 1: Likelihood of concordant expression (CE) as a function of coverage and differential haplotypic expression (DHE)

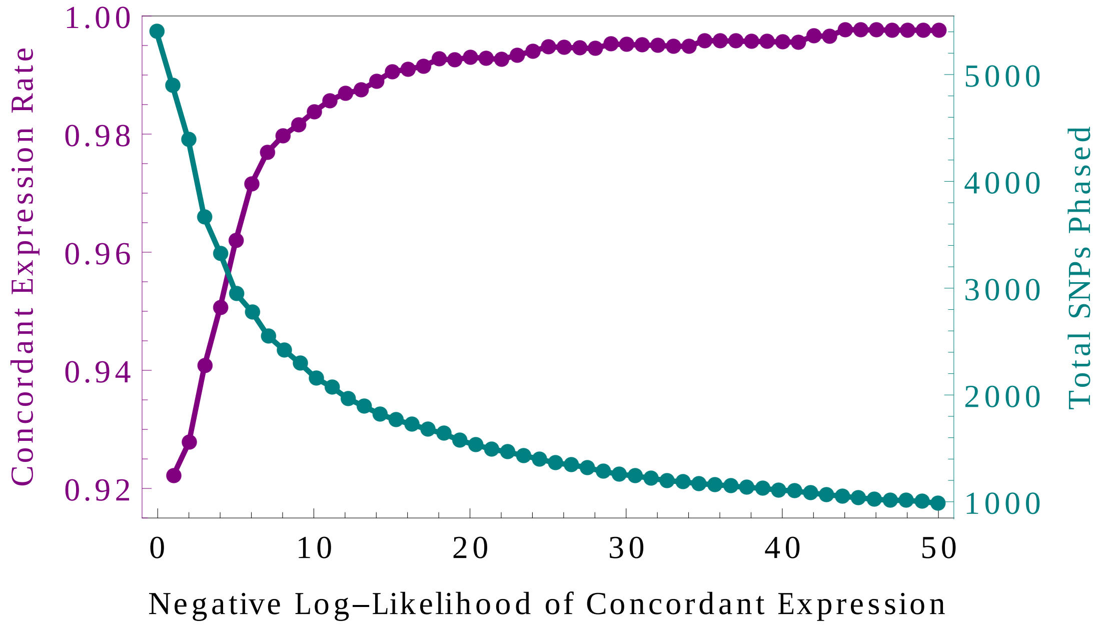

Supplementary Figure 2: Rate of concordantly expressed SNPs (purple) and total number of SNPs phased (green) by HapTree-X, as a function of λ, the negative log-likelihood of concordant expression.

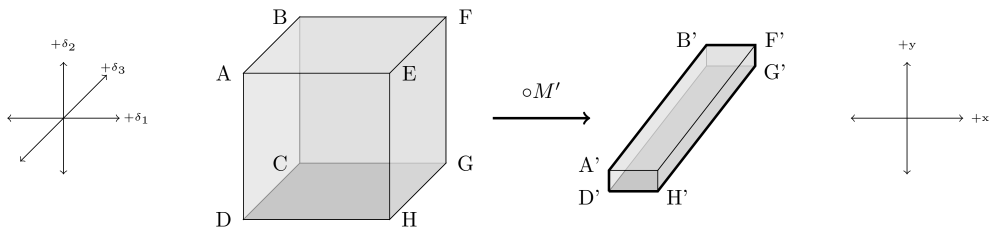

Supplementary Figure 3: Projection into 2-space of the cube Δ under the map: right multiplication by M'.

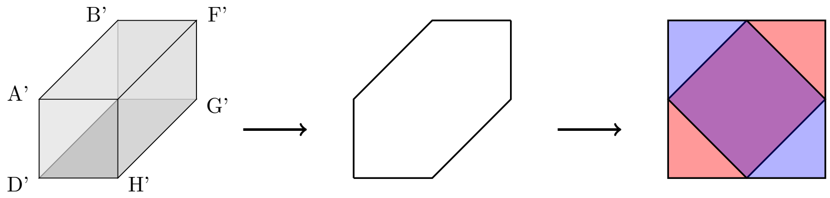

(a) $(\alpha_1, \alpha_2, \alpha_3) = (\frac{1}{3}, \frac{1}{3}, \frac{1}{3})$.

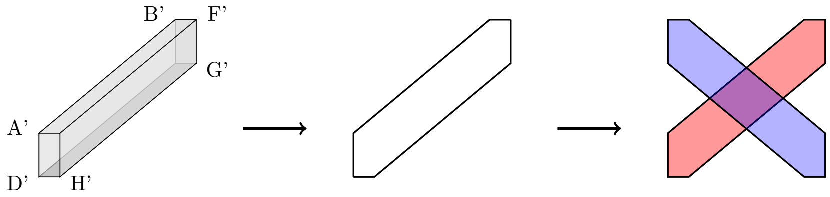

(b) $(\alpha_1, \alpha_2, \alpha_3) = (\frac{2}{20}, \frac{5}{20}, \frac{13}{20})$.

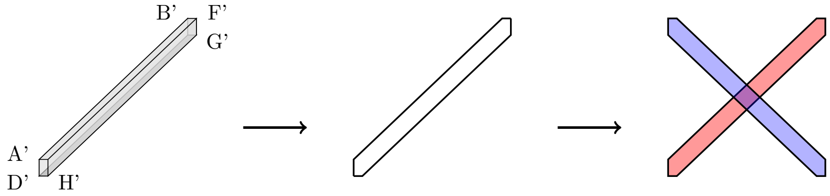

(c) $(\alpha_1, \alpha_2, \alpha_3) = (\frac{1}{20}, \frac{2}{20}, \frac{17}{20})$.

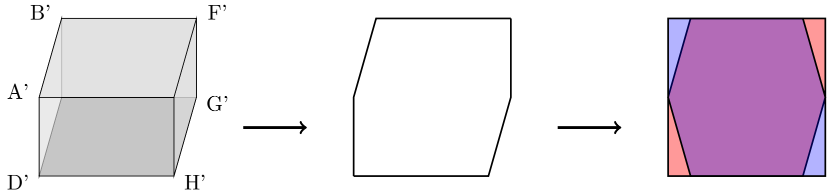

(d) $(\alpha_1, \alpha_2, \alpha_3) = (\frac{3}{4}, \frac{1}{8}, \frac{1}{8})$

Supplementary Figure 4: Examples of feasible regions for parallel phase in red, for switched in blue, and for both in purple for various values of $(\alpha_1, \alpha_2, \alpha_3)$.

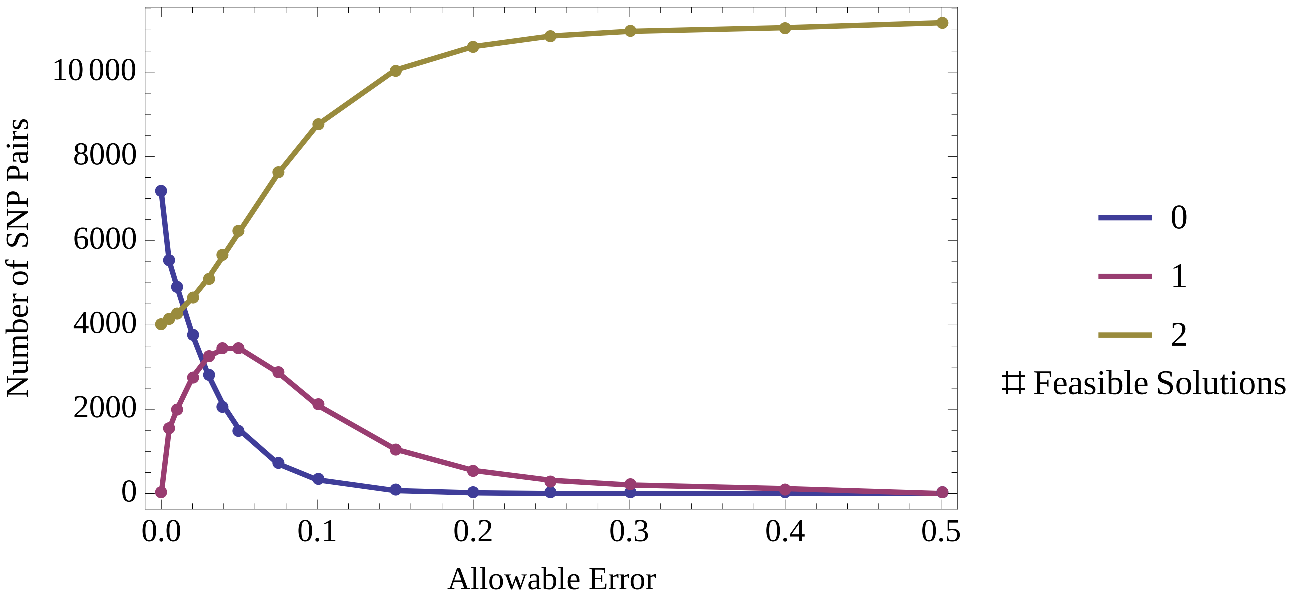

(a) Counts of SNP pairs with coverage at least 15 with two, one, and zero feasible solutions with varying allowed error for Poly-A- replicate 2

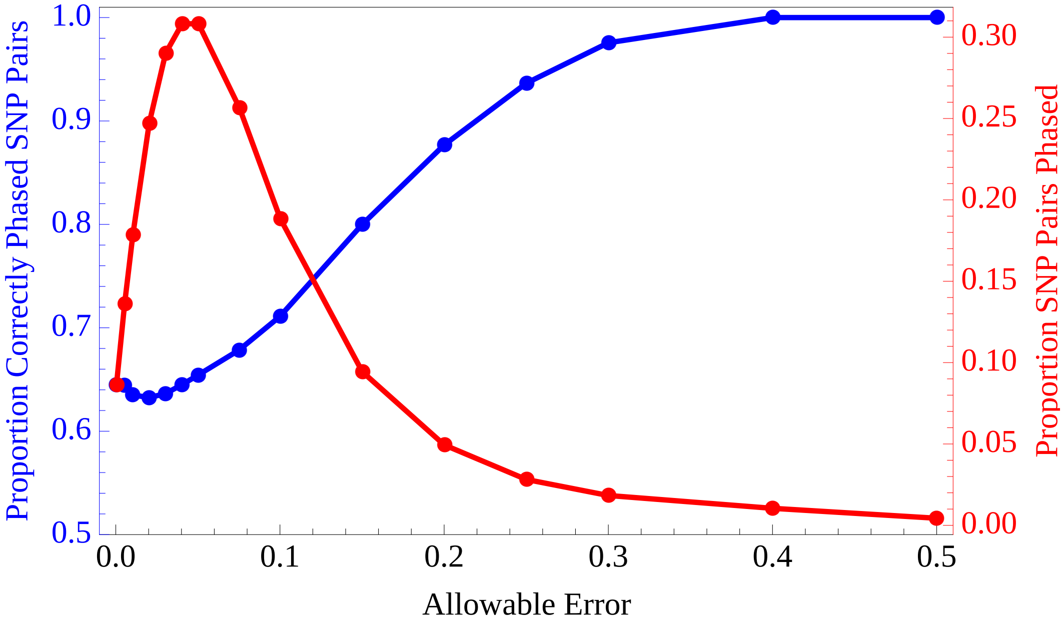

(b) Proportion of SNP pairs with a unique feasible solution (red) and the accuracy of that feasible solution (AUFS) (blue) compared against the platinum phased VCF with varying allowed error for Poly-A- replicate 2.

Supplementary Figure 5: For Poly-A- replicate 2 and varying allowed error, we report the counts of SNP pairs by number of feasible solutions, as well as the proportion of SNP pairs with a unique feasible solution and its accuracy.

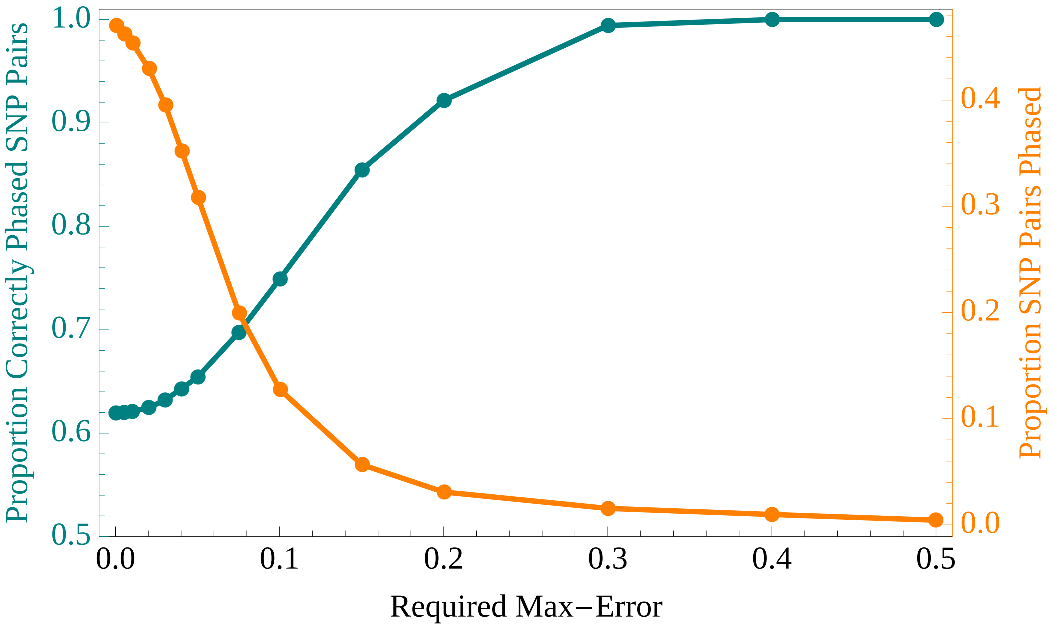

Supplementary Figure 6: For allowable min-error = 0.05 and varying required max-error ε_M , we report the proportion of accurately phased SNP pairs and the proportion of SNP pairs (out of all SNP pairs with coverage at least 15) able to be phased (that is, those with coverage at least 15, min-error $\leq$ 0.05, and max-error $\geq \varepsilon_M$.)

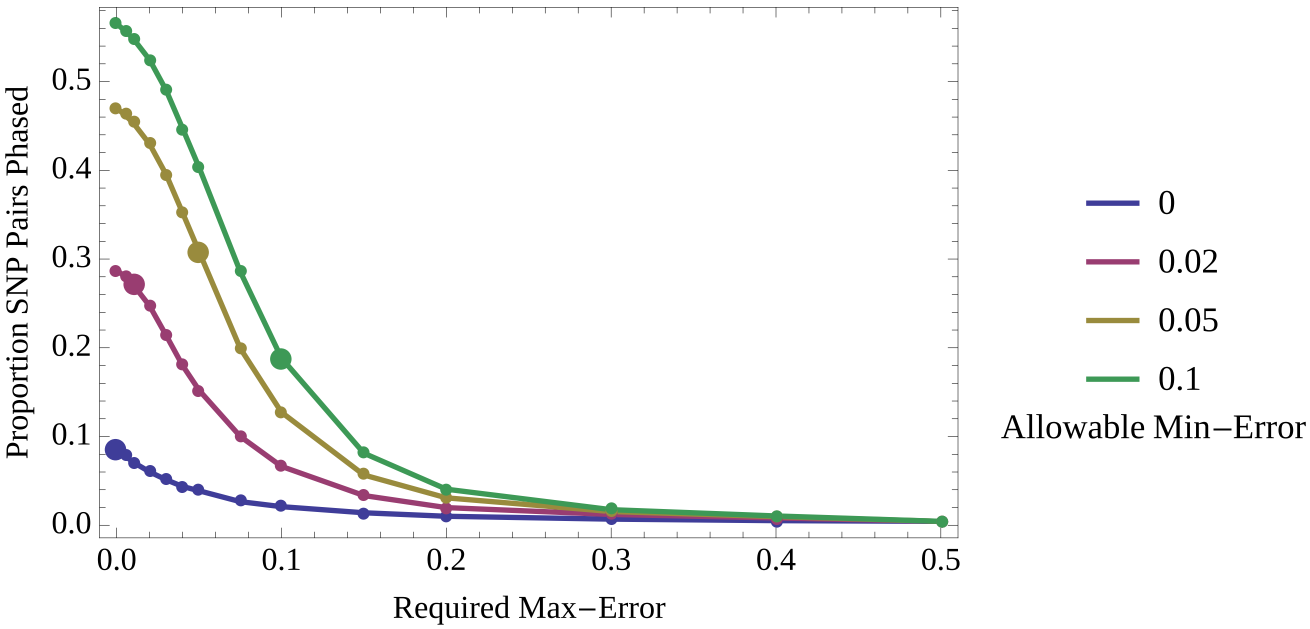

(a) The proportion of SNP pairs (out of all SNP pairs with coverage at least 15) able to be phased.

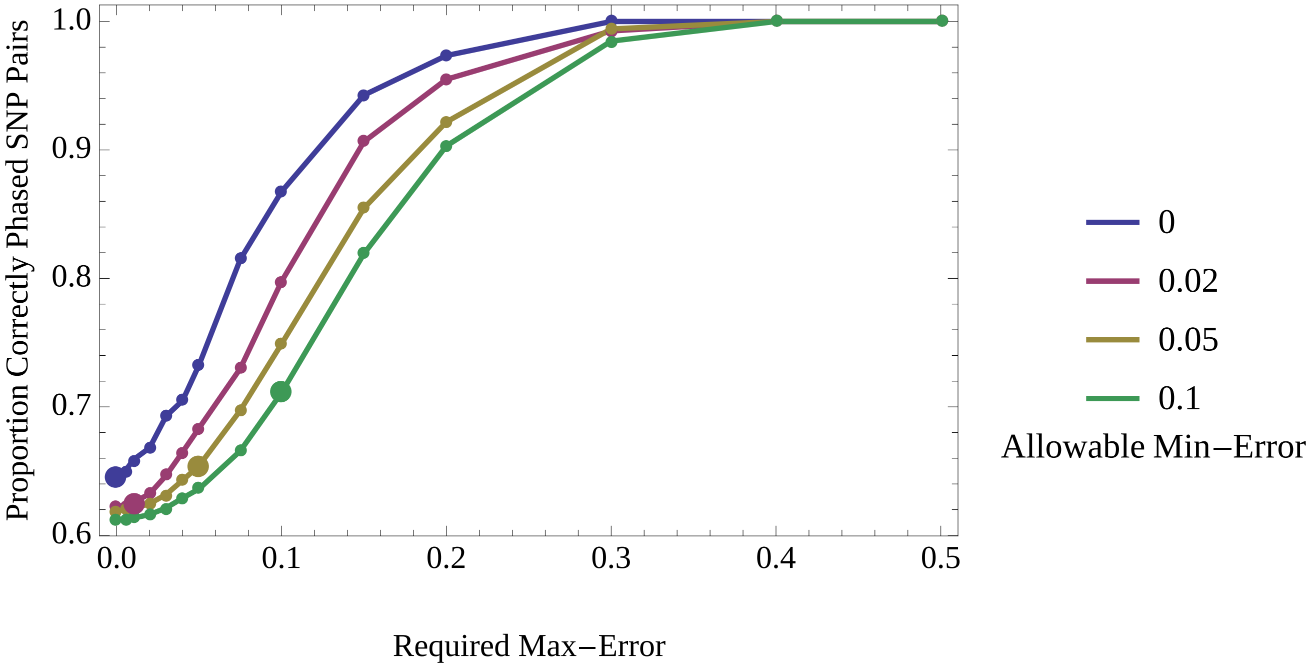

(b) Proportion of SNP pairs that were phased accurately relative to the gold-standard phased platinum VCF.

Supplementary Figure 7: For allowable min-error $\varepsilon_m \in [0, 0.02, 0.05, 0.1]$ (in purple, magenta, yellow, and green respectively) and varying required max-error ε_M we report the proportion of SNP pairs (out of all SNP pairs with coverage at least 15) able to be phased (a), as well as those which were accurately phased (b): The SNP pairs phased are those with coverage at least 15, min-error $\leq \varepsilon_m$, and max-error $\geq \varepsilon_M$.

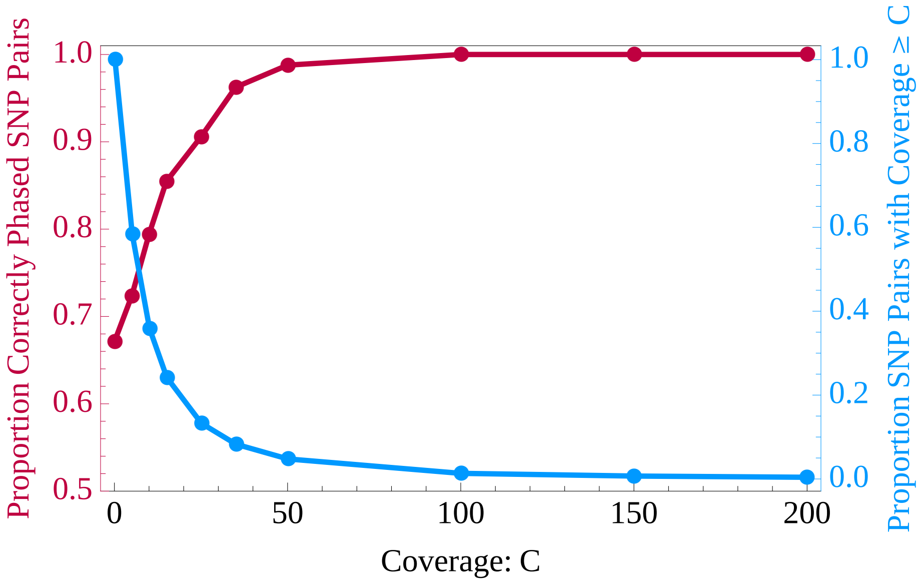

Supplementary Figure 8: Proportion of SNP pairs with coverage above a varying threshold (blue). Of those pairs, we phase those satisfying the error thresholds: $\varepsilon_m = 0.05$ and $\varepsilon_M = 0.15$; we report the proportion of correctly phased SNP pairs (red).

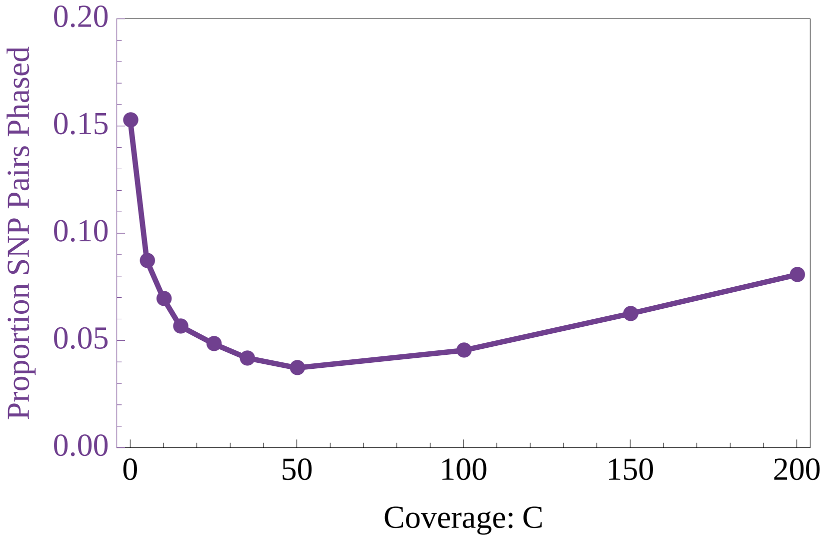

Supplementary Figure 9: Proportion of SNP pairs with coverage $\geq C$ which satisfy the error thresholds $\varepsilon_m = 0.05$ and $\varepsilon_M = 0.15$.

Supplementary References

[1] Shajii, A., Numanagić, I., Whelan, C. & Berger, B. Statistical binning for barcoded reads improves downstream analyses. *Cell Systems* **7**, 219 – 226.e5 (2018).

[2] Zhou, B. *et al.* Comprehensive, integrated, and phased whole-genome analysis of the primary encode cell line k562. *Genome research* **29**, 472–484 (2019).

[3] Trombetta, J. J. *et al.* Preparation of single-cell rna-seq libraries for next generation sequencing. *Current protocols in molecular biology* **107**, 4–22 (2014).

[4] Goods, B. A. *et al.* Blood handling and leukocyte isolation methods impact the global transcriptome of immune cells. *BMC immunology* **19**, 30 (2018).

[5] Roberts, A. & Pachter, L. Streaming fragment assignment for real-time analysis of sequencing experiments. *Nat Meth* **10**, 71–73 (2013). URL http://dx.doi.org/10.1038/nmeth.2251.

[6] Pachter, L. Models for transcript quantification from RNA-Seq. *arXiv preprint arXiv:1104.3889* (2011).

[7] De Concini, C. & Procesi, C. *Topics in Hyperplane Arrangements, Polytopes and Box-Splines*. 0172-5939 (Springer-Verlag New York, 2010).
