# Supplementary material for: Improved haplotype inference by exploiting long-range linking and allelic imbalance in RNA-seq datasets
Source: Nat Commun. 2020 Sep 16;11:4662. doi: 10.1038/s41467-020-18320-z (PMC7494856; doi:10.1038/s41467-020-18320-z)
Supplement: Supplementary file 3 — Reporting Summary [file 41467_2020_18320_MOESM3_ESM.pdf]

nature research

Corresponding author(s): Bonnie Berger
Ibrahim Numanagić

Last updated by author(s): Jul 3, 2020

Reporting Summary

Nature Research wishes to improve the reproducibility of the work that we publish. This form provides structure for consistency and transparency in reporting. For further information on Nature Research policies, see our Editorial Policies and the Editorial Policy Checklist.

Statistics

For all statistical analyses, confirm that the following items are present in the figure legend, table legend, main text, or Methods section.

n/a	Confirmed	
☐	☒	The exact sample size (*n*) for each experimental group/condition, given as a discrete number and unit of measurement
☒	☐	A statement on whether measurements were taken from distinct samples or whether the same sample was measured repeatedly
☒	☐	The statistical test(s) used AND whether they are one- or two-sided<br>*Only common tests should be described solely by name; describe more complex techniques in the Methods section.*
☒	☐	A description of all covariates tested
☒	☐	A description of any assumptions or corrections, such as tests of normality and adjustment for multiple comparisons
☒	☐	A full description of the statistical parameters including central tendency (e.g. means) or other basic estimates (e.g. regression coefficient) AND variation (e.g. standard deviation) or associated estimates of uncertainty (e.g. confidence intervals)
☒	☐	For null hypothesis testing, the test statistic (e.g. F, t, r) with confidence intervals, effect sizes, degrees of freedom and P value noted<br>*Give P values as exact values whenever suitable.*
☐	☒	For Bayesian analysis, information on the choice of priors and Markov chain Monte Carlo settings
☒	☐	For hierarchical and complex designs, identification of the appropriate level for tests and full reporting of outcomes
☒	☐	Estimates of effect sizes (e.g. Cohen's *d*, Pearson's *r*), indicating how they were calculated

Our web collection on statistics for biologists contains articles on many of the points above.

Software and code

Policy information about availability of computer code

Data collection	The full data collection report is available at http://haptreex.csail.mit.edu/ and<br>https://github.com/0xTCG/haptreex/blob/master/paper/experiments.ipynb
Data analysis	The full data analysis report that includes the data links and the software versions is available at http://haptreex.csail.mit.edu/ and<br>https://github.com/0xTCG/haptreex/blob/master/paper/experiments.ipynb.<br>The following software versions were used to conduct the experiments:<br>- HapTree-X v2.0<br>- HapCUT2 v1.2 (de33b57)<br>- HapCUT v0.7 (254c473)<br>- phASER v1.1.1 (20eb3b)<br>- GATK v4 (v3.8 for calling 10X VCFs)<br>- EMA v0.6.2.

For manuscripts utilizing custom algorithms or software that are central to the research but not yet described in published literature, software must be made available to editors and reviewers. We strongly encourage code deposition in a community repository (e.g. GitHub). See the Nature Research guidelines for submitting code & software for further information.

Data

Policy information about availability of data

All manuscripts must include a data availability statement. This statement should provide the following information, where applicable:

- Accession codes, unique identifiers, or web links for publicly available datasets
- A list of figures that have associated raw data
- A description of any restrictions on data availability

The HapTree-X software is free and open source and is available at http://haptreex.csail.mit.edu.

The RNA-seq sequencing data for GM12878 (nucleus, cytosol and whole) and K52 cell lines is available through ENCODE project (track wgEncodeCshlLongRnaSeq). 10X samples (NA12878 and NA24385) are available from 10X Genomics de novo Assembly collection (Supernova 2.0.0; https://www.10xgenomics.com/resources/datasets/). Whole exome data is available in BAM format through 1000 Genomes Phase 3 (ID: NA12878, version: 20121211). The GIAB RNA-seq data (NA12878, NA24143, NA24219, NA24385 and NA24631) is available for download at http://haptreex.csail.mit.edu/datasets. NA12878 WGS sample (BAM and VCF) is available through Illumina Platinum Genomes project (gs://genomics-public-data/platinum-genomes).
The validation VCFs datasets are available through the Genome in the Bottle project (https://github.com/genome-in-a-bottle/giab_latest_release).
GEUVADIS samples are available through 1000 Genomes project; the exact accession IDs are: ERR188033, ERR188080, ERR188150, ERR188246, ERR188312, ERR188340, ERR188379, ERR188426, ERR188036, ERR188091, ERR188185, ERR188262, ERR188314, ERR188361, ERR188395, ERR188435, ERR188043, ERR188146, ERR188213, ERR188264, ERR188335, ERR188368, ERR188397, ERR188076, ERR188149, ERR188215, ERR188277, ERR188337, ERR188371 and ERR188418.

The exact download links for all datasets are available in the Jupyter Notebook format at http://haptreex.csail.mit.edu and https://github.com/0xTCG/haptreex.

Field-specific reporting

Please select the one below that is the best fit for your research. If you are not sure, read the appropriate sections before making your selection.

☒ Life sciences ☐ Behavioural & social sciences ☐ Ecological, evolutionary & environmental sciences

For a reference copy of the document with all sections, see nature.com/documents/nr-reporting-summary-flat.pdf

Life sciences study design

All studies must disclose on these points even when the disclosure is negative.

Sample size	We evaluated all methods on 6 individual genomes: 5 from the well-established benchmark GIAB dataset that covers three diverse genetic backgrounds, and a K562 cell line. These genomes were selected due to the availability of the ground truth phasing data (to the best of our knowledge, there is no well-established validated ground truth phasing data for other publicly available samples).<br>We evaluated the phasing performance on GIAB genomes— in particular NA12878— on wide variety of sequencing technologies, including RNA-seq, Illumina WGX, Illumina WXS and 10X Genomics. NA12787 RNA-seq performance was furthermore validated on 4 different RNA-seq sequencing modes: in-house, nucleus, cytosol, and whole cell to account for the differences in RNA-seq experimental design and varying sequencing coverage.<br>Finally, we evaluated the effect of DASE phasing on additional 30 publicly available GEUVADIS samples that were selected to cover as many different ethnic backgrounds as possible to avoid any hereditary bias.
Data exclusions	No data was excluded from the study.
Replication	We ran the software repeatedly on multiple platforms (macOS, Linux). We successfully replicated the reported results on all platforms.
Randomization	Randomization was not necessary because each evaluated tool operated on a single sample. Samples were grouped and compared based on their cell line and the underlying sequencing technology.
Blinding	Blinding was not necessary because each evaluated tool operated on a single sample only.

Reporting for specific materials, systems and methods

We require information from authors about some types of materials, experimental systems and methods used in many studies. Here, indicate whether each material, system or method listed is relevant to your study. If you are not sure if a list item applies to your research, read the appropriate section before selecting a response.

Materials & experimental systems

n/a	Involved in the study
☒	☐ Antibodies
☐	☒ Eukaryotic cell lines
☒	☐ Palaeontology and archaeology
☒	☐ Animals and other organisms
☒	☐ Human research participants
☒	☐ Clinical data
☒	☐ Dual use research of concern

Methods

n/a	Involved in the study
☒	☐ ChIP-seq
☒	☐ Flow cytometry
☒	☐ MRI-based neuroimaging

Eukaryotic cell lines

Policy information about cell lines

Cell line source(s)	Coriell (GIAB); ENCODE/Cold Spring Harbor Lab (K562, GM12878)
Authentication	None (detailed sequencing information is available in Supplementary Note 2)
Mycoplasma contamination	Not tested
Commonly misidentified lines (See ICLAC register)	No commonly misidentified line was used in this study.
