# Supplementary material for: Improved haplotype inference by exploiting long-range linking and allelic imbalance in RNA-seq datasets
Source: Nat Commun. 2020 Sep 16;11:4662. doi: 10.1038/s41467-020-18320-z (PMC7494856; doi:10.1038/s41467-020-18320-z)
Supplement: Supplementary file 2 — Peer Review File [file 41467_2020_18320_MOESM2_ESM.docx]

Peer Review Information

**Journal:** Nature Communications

**Manuscript title:** Improved haplotype inference by exploiting long-range linking and allelic imbalance in RNA-seq datasets

**Corresponding author name(s):** Dr Bonnie Berger and Dr Ibrahim Numanagic

**Editorial notes:**

**Transferred manuscripts**

This manuscript has been previously reviewed at another journal that is not operating a transparent peer review scheme. This document only contains reviewer comments and rebuttal letters for versions considered at *Nature Communications*.

**Reviewer comments & decisions:**

**Reviewer comments, first version:**

Reviewer #1 (Remarks to the Author):


The authors have significantly revised the manuscript in response to the previous review. The results presented in Supplementary Table 3-4 indicate that the DASE method phases 2-3% additional SNPs relative to exome-seq/RNA-seq alone albeit with a higher switch error rate. In addition, Supplementary Table 9 lists variants phased by DASE in disease associated genes. Additional results on 10X and RNA-seq data show that HapTree-X either outperforms other read-based phasing tools or has similar performance. My comments below are mainly about the practical utility of the DASE based phasing since that is the novel methodological contibution of this paper.


1. RNA-seq provides a direct source of haplotype information where reads that span introns can link together variants present far away from each other on different exons. This was the focus of the PHASER paper published in Nature Communications, 2017. The DASE method uses an "indirect" source of haplotype information (allele-specific expression) to phase variants in human genomes. This is analogous to population-based phasing methods which use patterns of LD in a population to infer the most likely haplotypes. However, the key difference is population-based phasing can phase the vast majority of common variants with high accuracy while DASE adds a small fraction of SNPs to the phasing using sequence reads.

2. The discussion is very brief (one paragraph). In the last review, the comments about the limitations of using the DASE approach for phasing using RNA-seq data should be addressed in the discussion.
Similarly, the implications of this method for disease studies should also be discussed. In the response to the reviewer document, the authors mention that "there are certain advantages with our RNA/DASE-based approach that alleviates the reviewer’s concerns, which we now add to Discussion", but I did not see this in the Discussion.

3. As the authors pointed out in response to a comment by a Reviewer, the main difference between HapTree and HapTree-X is the DASE probabilistic model. HapTree-X seems to have the same or slightly better performance that competing tools on 10X data. Do the results from using HapTree-X differ from HapTree on 10X data or other genomic datasets? Also, would adding the DASE information improve the phasing when combined with Illumina whole-genome sequencing?


Minor comments:


In Supp Table 13, why is the switch error rate for two of the genomes so high?

"Though HapCUT2 exhibits a better memory usage, HapTree-X achieves better accuracy than HapCUT2 at a still feasible memory usage (Figure 2).": I don't think that this statement should be in legend of Table 2.


1.4.2 RNA-seq only resulta -> RNA-seq only results

The HapTree-X software is not open source. Please mention if there are restrictions on usage for academic/commercial users.


Reviewer #3 (Remarks to the Author):

The presentation of the methods and results in the manuscript have been improved, especially by including the additional GIAB samples and by making the software available. However, my original concerns of the potential impact of the paper remains. While this is an elegant theoretical framework that shows how allelic imbalances in expression can in principle be used to improve phasing, in practice the improvements are extremely modest (<10% improvement to phase block length). The authors add a new supplemental table 9 of the specific genes that were improved, but list only 7 examples, and many of which were improvements to phasing of a single SNP. In its current form I would recommend the manuscript for a more specialized journal, especially since there are no new biological results. The authors could perhaps address my concern by applying the method to a large number of samples and reporting on the frequency of compound heterozygosity (or a similar study) to highlight how this method could be used in practice.

**Author rebuttal, first version:**

Reviewer #1

The authors have significantly revised the manuscript in response to the previous review. The results presented in Supplementary Table 3-4 indicate that the DASE method phases 2-3% additional SNPs relative to exome-seq/RNA-seq alone albeit with a higher switch error rate. In addition, Supplementary Table 9 lists variants phased by DASE in disease associated genes. Additional results on 10X and RNA-seq data show that HapTree-X either outperforms other read-based phasing tools or has similar performance. My comments below are mainly about the practical utility of the DASE based phasing since that is the novel methodological contribution of this paper.

Thank you for your comments!

In order to address the concerns about the usability of DASE, we have greatly improved our software and have completely redone our experimental pipeline. By doing so, we have fixed many of the inconsistencies found in the old pipeline (details are described in our reply to the second reviewer). The new results demonstrate much stronger impact of DASE on phasing quality (improvements in phased block span now often exceed 10%, and in one case exceeds 25%). For more details, please see the Results section, paragraph **Effect of DASE** .

1. RNA-seq provides a direct source of haplotype information where reads that span introns can link together variants present far away from each other on different exons. This was the focus of the PHASER paper published in Nature Communications, 2017. The DASE method uses an "indirect" source of haplotype information (allele-specific expression) to phase variants in human genomes. This is analogous to population-based phasing methods which use patterns of LD in a population to infer the most likely haplotypes. However, the key difference is population-based phasing can phase the vast majority of common variants with high accuracy while DASE adds a small fraction of SNPs to the phasing using sequence reads.

We agree that the population-based phasing can have higher accuracy over DASE if we consider only common variants (we have already noted this in our original RECOMB 2015 extended abstract and oral presentation on Haptree-X). However, please note that it is not always possible to perform population-based phasing due to either lack of a control population or of computational resources (panel construction might take days even with a few samples). Even if possible, population-based phasing often misses rare variants that HapTree-X can find. Finally, note that the DASE-based phasing requires only RNA-seq data and costs almost nothing (the additional runtime cost is negligible)— as such, it can be incorporated as a pre- or post-processing step in addition to population-based phasing (as noted in Discussion).

To illustrate our point, we have included here an example of phases produced by EAGLE, a state-of-the-art population based phaser, on GM12878 RNA-seq whole-cell data with the 1000 Genomes Phase 3 haplotypes as a reference panel. As seen on the right, EAGLE cannot phase many rare variants despite having NA12878 in the reference panel; however, a joint EAGLE & HapTree-X pipeline would more completely phase these genes.

2. The discussion is very brief (one paragraph). In the last review, the comments about the limitations of using the DASE approach for phasing using RNA-seq data should be addressed in the discussion. Similarly, the implications of this method for disease studies should also be discussed. In the response to the reviewer document, the authors mention that "there are certain advantages with our RNA/DASE-based approach that alleviates the reviewer’s concerns, which we now add to Discussion", but I did not see this in the Discussion.

We have expanded the Discussion and included the missing parts as well as others.

3. As the authors pointed out in response to a comment by a Reviewer, the main difference between HapTree and HapTree-X is the DASE probabilistic model. HapTree-X seems to have the same or slightly better performance that competing tools on 10X data. Do the results from using HapTree-X differ from HapTree on 10X data or other genomic datasets? Also, would adding the DASE information improve the phasing when combined with Illumina whole-genome sequencing?

Unlike HapTree-X, HapTree does not scale and is unable to analyze large datasets. As a result, we were not able to evaluate it on our datasets (especially on 10X datasets due to their size). HapTree-X ships with many novel improvements that significantly reduce redundant computations in the original algorithm; we now make this point clearer in the main manuscript ( **Introduction** ) Moreover, we have now ported the remaining HapTree features to HapTree-X (most notably polyploid phasing), which means that the latest HapTree-X is a complete replacement for HapTree (and of course, HapTree-X can additionally phase large data samples and long linked read data).

RNA-seq data can indeed be used to improve the WGS phasing (thanks for the suggestion!), and we have added the relevant experiment to the paper (paragraph **Other technologies** and **Table 4** ). While HapTree-X phases this sample better than HapCUT2, even without RNA-seq data, the incorporation of RNA-seq data increases the number of phased SNPs by roughly 4,000 while the total length of phased blocks increased by 60 MB (30% overall improvement). The effect of DASE itself was more modest as compared to its effect in joint exome data phasing: here, DASE added a couple hundred SNPs and increased the total phased block length by a couple hundred kilobases. The effect of DASE on the overall phasing speed was negligible (for the record, the whole joint phasing process on two 140GB and 40GB BAMs took around twenty minutes with 4 threads on an iMac desktop computer— other tools are not even able to parse these files in that time!).

**Minor comments:**

— In Supp Table 13, why is the switch error rate for two of the genomes so high?

We believe that this was due to the issues with ground truth data— all phasers report similar spikes in switch errors, and those samples are not as well-studied as the canonical NA12878

sample. Note that we have removed Supplementary Table 13 in the latest version of the manuscript and have integrated the relevant results into Table 3.

— "Though HapCUT2 exhibits a better memory usage, HapTree-X achieves better accuracy than HapCUT2 at a still feasible memory usage (Figure 2).": I don't think that this statement should be in legend of Table 2.

We have removed this statement.

— 1.4.2 RNA-seq only resulta -> RNA-seq only results

Corrected— thank you!

— The HapTree-X software is not open source. Please mention if there are restrictions on usage for academic/commercial users.

Apologies for not putting the source code online. HapTree-X is an open source package, and the source code can be found at [http://haptreex.csail.mit.edu](http://haptreex.csail.mit.edu/) .

Reviewer #3

The presentation of the methods and results in the manuscript have been improved, especially by including the additional GIAB samples and by making the software available. However, my original concerns of the potential impact of the paper remains. While this is an elegant theoretical framework that shows how allelic imbalances in expression can in principle be used to improve phasing, in practice the improvements are extremely modest (<10% improvement to phase block length). The authors add a new supplemental table 9 of the specific genes that were improved, but list only 7 examples, and many of which were improvements to phasing of a single SNP. In its current form I would recommend the manuscript for a more specialized journal, especially since there are no new biological results. The authors could perhaps address my concern by applying the method to a large number of samples and reporting on the frequency of compound heterozygosity (or a similar study) to highlight how this method could be used in practice.

We have completely revamped and enhanced the paper, including additional experiments, methodological enhancements, and even more biological results. We hope these changes will now be sufficient enough to warrant publication in Nature Communications. We would like to thank the reviewer for their comment— further investigation led us to an experimental mistake on our part that had a significant impact on the results we presented before: we mistakenly fed the same set of incorrect VCF files consistently to all phasers. To fix this, we have generated a separate VCF file for each sample by running GATK's Best Practices pipeline for RNA-seq, 10X and exome data (as appropriate), and have used more robust GIAB phased VCFs for validating

our calls. Finally, we reran all tools with the appropriate VCF files (technical details can be found in the experimental notebook on our website: [http://haptreex.csail.mit.edu](http://haptreex.csail.mit.edu/) ). As a result of these changes:

- The increase in the total length of phased blocks due to DASE-based phasing ranges from a few percent in case of low coverage GIAB samples, 10–15% in joint exome & RNA-seq datasets, and up to 25% in the K562 leukemia cell line. These results are consistent with the theory behind DASE as outlined in Supplementary Materials ( **section 1** ) and Discussion: more coverage provides better DASE performance (the in-house sequenced samples where DASE improvements were modest had extremely low coverage, and many genes were not covered at all). DASE also helps HapTree-X to phase way more SNPs than previously reported (up to 1,000 SNPs in some samples as compared to HapTree-X without DASE).
- Please note that, theoretically speaking, the total length of phased blocks cannot be radically increased even if we had a hypothetical "perfect" phaser due to the nature of RNA-seq data: the transcriptome is nearly 40x shorter than the genome, and the DASE-targeted blocks are restricted to gene units. The fact that HapTree-X can consistently outperform other phasers on a wide variety of samples, and phase more variants (and furthermore improve phasing in disease-related genes, as shown in the paper) with no impact on computational resources shows that DASE is useful (see Discussion).
- We certainly agree that it is hard to see the utility of a method when benchmarked on a single individual. Thus, we ran HapTree-X and other tools on thirty different GEUVADIS samples from diverse backgrounds. Note that GEUVADIS is a low-coverage dataset, but was the only large public dataset that we could access in a timely manner for this revision. Nevertheless, we observed that HapTree-X consistently outperforms other tools, and that it provides better phases in longer genes that have disease associations: Figure 3 graphically shows the consistent phasing improvements in the *BCR* gene (we observed these improvements in 17 of 30 samples).
- Finally, note that the aforementioned GEUVADIS samples are all healthy human samples, and as such lack many disease markers. Furthermore, we do not possess the ground truth data for these samples. We would expect to see even stronger results in patient cohorts— this intuition is partly validated by the fact that the K562 leukemia cell line benefited most from DASE. We certainly plan to further explore such datasets in the future (we outlined this in the updated Discussion). But others can certainly run Haptree-X on such datasets! Furthermore, a more detailed analysis of patient cohorts requires significant time and resource commitments that are outside the scope of this study.

**Reviewer comments, second version:**

REVIEWERS' COMMENTS:

Reviewer #1 (Remarks to the Author):
The authors have substantially revised the manuscript. The response to the comments from the previous review are satisfactory. The software is now available and open source.

Minor comments:

Table 2: legend for the columns would be useful.

Table 2: the switch error rate for NA24149 is very high. As pointed out in the previous review, using genomes/datasets for which high quality phased haplotype information is not available, is not recommended.

Figure 2 and 3: reference genome used for the positions of the variants should be indicated

The reference from Bioinformatics 2018 (https://pubmed.ncbi.nlm.nih.gov/29950014/) should be referenced in the discussion on single cell phasing.


Reviewer #3 (Remarks to the Author):
The revised manuscript satisfies my concerns. I’m pleased to see the corrected experiments demonstrate a more substantial improvement in phase block length and scalability.

**Author rebuttal, second version:**

Reviewer #1

The authors have substantially revised the manuscript. The response to the comments from the previous review are satisfactory. The software is now available and open source.

We would like to thank the reviewer for reviewing our manuscript!
**Minor comments:**

— Table 2: legend for the columns would be useful.
Added.

— Table 2: the switch error rate for NA24149 is very high. As pointed out in the previous review, using genomes/datasets for which high quality phased haplotype information is not available, is not recommended.

We have removed the NA24149 sample from the consideration.

— Figure 2 and 3: reference genome used for the positions of the variants should be indicated Added.

— The reference from Bioinformatics 2018 (<https://pubmed.ncbi.nlm.nih.gov/29950014/)> should

be referenced in the discussion on single cell phasing.

Added.

Reviewer #3

The revised manuscript satisfies my concerns. I’m pleased to see the corrected experiments demonstrate a more substantial improvement in phase block length and scalability.

We would like to thank the reviewer for reviewing our manuscript!

**Reviewer comments, third version:**

**Author rebuttal, third version:**
